# RNA and the RNA-binding protein FUS act in concert to prevent TDP-43 spatial segregation

**DOI:** 10.1016/j.jbc.2024.105716

**Published:** 2024-02-02

**Authors:** Clément Demongin, Samuel Tranier, Vandana Joshi, Léa Ceschi, Bénédicte Desforges, David Pastré, Loic Hamon

**Affiliations:** 1 SABNP, Univ Evry, INSERM, U1204, Université Paris-Saclay, Evry, France; 2 Institut de Pharmacologie et de Biologie Structurale, IPBS, Université de Toulouse, CNRS, UPS, Toulouse, France

**Keywords:** RNA-binding protein, RNA, compartmentalization, protein solubilization, protein aggregation, low complexity domain, neurodegenerative diseases

## Abstract

FUS and TDP-43 are two self-adhesive aggregation-prone mRNA-binding proteins whose pathological mutations have been linked to neurodegeneration. While TDP-43 and FUS form reversible mRNA-rich compartments in the nucleus, pathological mutations promote their respective cytoplasmic aggregation in neurons with no apparent link between the two proteins except their intertwined function in mRNA processing. By combining analyses in cellular context and at high resolution *in vitro*, we unraveled that TDP-43 is specifically recruited in FUS assemblies to form TDP-43–rich subcompartments but without reciprocity. The presence of mRNA provides an additional scaffold to promote the mixing between TDP-43 and FUS. Accordingly, we also found that the pathological truncated form of TDP-43, TDP-25, which has an impaired RNA-binding ability, no longer mixes with FUS. Together, these results suggest that the binding of FUS along nascent mRNAs enables TDP-43, which is highly aggregation-prone, to mix with FUS phase to form mRNA-rich subcompartments. A functional link between FUS and TDP-43 may explain their common implication in amyotrophic lateral sclerosis.

TDP-43 and FUS have been under scrutiny over the past years due to their link with amyotrophic lateral sclerosis (ALS) and frontotemporal lobar degeneration (FTLD). Both FUS and TDP-43 are nuclear proteins that assemble into insoluble cytoplasmic aggregates in the neurons of patients affected by these two incurable neurodegenerative diseases (1, 2, 3, 4). They also share similar targets and structure, both proteins being associated to mRNA and harboring long unstructured self-adhesive domains to regulate their higher order assemblies. A tight regulation of dynamic assemblies of TDP-43 and FUS is required to fulfill their intertwined functions associated with the mRNA life cycle including transcriptional regulation, pre-mRNA splicing, mRNA localization, and processing (5, 6, 7). To enable reversibility of these assemblies, TDP-43 and FUS should remain soluble which is a challenging task for cells due to the presence of self-adhesive low complexity domains (LCD) prone to aggregation. The solubility of TDP-43 and FUS is also controlled by their binding to RNA, which is their main partner *in vivo*. Indeed, a high RNA/RBP molar ratio favors the dynamics and reversible assemblies of TDP-43 and FUS in the nucleus under physiological conditions (8, 9). A high RNA/RBP molar ratio could be also observed in the cytoplasm in mRNA-rich stress granules (SGs) after acute cellular stress (10). The presence of TDP-43 and FUS in SGs may indeed preserve their solubility (9, 11). Conversely, a low RNA/RBP ratio promotes multivalent protein–protein interactions, mainly *via* LCDs, and leads to the formation of insoluble aggregates. In an alternative model, SGs can be considered as crucibles in the formation of cytoplasmic inclusions linked to neurodegenerative diseases (12, 13) due to the high concentration of LCD-rich proteins that promotes the formation of an aggregation-prone subcompartment. Again in agreement with a higher protein solubility in the presence of mRNA, an altered binding between these proteins and RNA, in particular through pathological mutations, can lead to their aggregation (14, 15).

Their intertwined functions in the regulation of transcription and in mRNA splicing lead TDP-43 and FUS to associate with approximately 30% of the transcriptome in the human brain (16). However, their binding sites on mRNAs, as observed by cross-linking and immunoprecipitation methods, seem quite distinct (17, 18). FUS binds nonspecifically along nascent mRNAs (19) while TDP-43 forms clusters in GU-rich sequences. Despites these differences, the sets of genes with altered expression levels upon TDP-43 or FUS knockdown exhibit significantly overlapping transcriptome profile (20). In addition, their coordination is necessary to regulate the expression of common targets such as histone deacetylase 6 (21). In animal models like *drosophila* and zebrafish, knockdown, overexpression, and rescue studies of TDP-43 and FUS support the notion that TDP-43 and FUS participate in mRNA maturation pathways (22, 23, 24). Beyond mRNA-related functions, FUS and TDP-43 also have common protein partners. For example, both of them have the ability to stall RNA PolII processing (25, 26). Finally, co-immunoprecipitation studies demonstrate that although these two proteins could be present in their mutual interactome (27, 28, 29), they almost never coexist in the cytoplasmic aggregates in the neurons of ALS/FTLD patients (30, 31). There are thus numerous lines of evidence indicating that the simultaneous presence of FUS and TDP-43 on the same RNA substrates is required for them to perform similar or complementary biological functions.

We propose to study the primary relationship between these two RBPs, particularly their interplay for the binding to mRNA and the consequence of these interactions in their ability to form compartments in which they could either mix or demix. Our working hypothesis is based on the fact that despite a low specificity for RNA sequences (19, 32), FUS has a preference for RNA structures (33, 34) and can remodel RNA following interaction with its RGG domains (35). Through these characteristics, FUS binding to nascent mRNAs could form FUS-rich compartments in which TDP-43 recruitment and binding to intron-rich GU sequences could occur with high affinity *via* TDP-43’s RNA-recognition motifs (RRM) (17, 36, 37). The binding of TDP-43 to RNA is cooperative, which, along with its self-attracting N-terminal domain, can secure the formation of TDP-43–rich compartments on GU-rich sequences on long RNAs (38). Here, we combine analyzes in a cellular context using microtubules as nano-platforms (39) with structural information obtained at the single molecule level (40) to gain access to the spatial organization of TDP-43 and FUS. We first demonstrate that TDP-43 and FUS interact with each other. However, TDP-43 is soluble in FUS-rich compartment but the reverse is not observed. We have evidenced that the partial miscibility between FUS and TDP-43 is enhanced *in vitro* by the presence of RNA. At low TDP-43 concentration, we observe a homogenous distribution of TDP-43 along RNA, whereas at high concentration, TDP-43 forms subcompartments still associated with FUS. We also demonstrate that the colocalization of FUS and TDP-43 in SGs is more pronounced than with other RBPs but when TDP-43 loses its ability to bind cooperatively to RNA, the colocalization is affected. Finally, we observe that the RNA-binding capacity of TDP-43 is essential to preserve the miscibility of TDP-43 with FUS. Consistently, pathological truncations of TDP-43 having lost all or part of the RRMs are excluded from the FUS–RNA complexes and aggregate independently of FUS. Therefore, the interaction between these two ALS-linked proteins can significantly contribute to their essential functions in RNA metabolism. Importantly, TDP-43/FUS interaction may help TDP-43 by preventing it from forming distinct assemblies on its own, which would constitute an early step towards TDP-43 aggregation.

## Results

### Among a set of RBPs, TDP-43 and FUS have the best colocalization score

**Figure 1:**
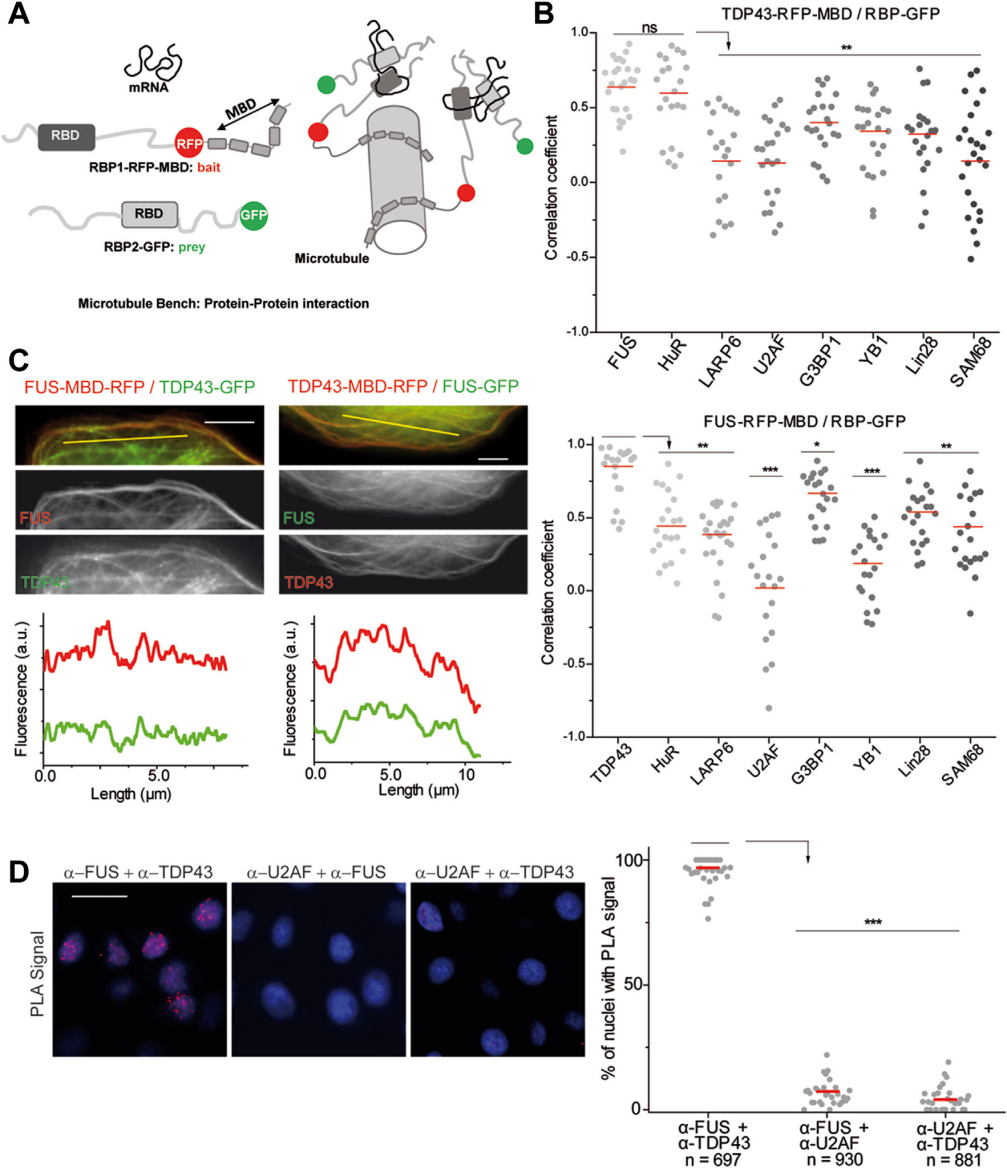
**TDP-43 and FUS interact in the cellular context.***A*, schematic representation of the microtubule bench assay principle. In brief, a protein of interest (bait) fused to microtubule-associated domains of Tau (MBD) and RFP (or GFP) is brought onto microtubules in living cells whereas the presence of a GFP- (or RFP-fused) protein partner (prey) on microtubules reveals the interaction by colocalization of the fluorescence signals on microtubules. *B*, scatter plot representing the colocalization level of MBD-fused TDP-43 (*upper* panel) or MBD-fused FUS (*lower* panel) with one of the eight tested RBPs. Each data point represents a correlation coefficient between fluorescence intensities from *red* and *green* channels along a line crossing the microtubules. The plot shows the data from two independent experiments. *Red* lines show mean values. Significances between correlation coefficients were obtained using *t* test; ∗*p* < 0.05; ∗∗*p* < 0.01; ∗∗∗*p* < 0.005; ns, not significant. *C*, U2OS cells were cotransfected with the constructions encoding FUS (*left* panel) or TDP-43 (*right* panel) fused to RFP-MBD and the plasmid expressing full length TDP-43 (*left* panel) or FUS (*right* panel) fused to GFP. The fluorescence intensities from the two channels along the *yellow* lines are shown below in the respective microphotographs. Scale bar represents 3 μm. *D*, *left* panel: Representative images for proximity ligation assay (PLA) revealing the colocalization of TDP-43 and FUS in HeLa cells. Cells were fixed, incubated with anti-FUS, anti U2AF65, or anti-TDP-43 antibodies, and with nucleotide probes, ligase, and polymerase as described in the Experimental procedures section. Scale bar represents 20 μm. *Right* panel: scatter plot representing the percentage of nuclei with a PLA signal. Each point corresponds to 30 cells analyzed. n: number of cells analyzed. *Red* lines show mean values. ∗∗∗*p* < 0.005; *t* test.

To determine whether two proteins interact with each other in the cytoplasm, the microtubule network was used as an intracellular bench (41). A fusion protein containing an RFP tag and a microtubule-binding domain was overexpressed in cells to serve as a bait. In parallel, these cells were modified to overexpress a prey protein with a GFP tag (Fig. 1*A*). Colocalization of prey protein with the microtubule network in cells expressing the bait protein would indicate that the two proteins interact with each other either directly or indirectly (Fig. S1). One of the strengths of this analysis is that it reveals interactions in a cellular context, without any cell lysis or protein purification. Interaction between TDP-43 and FUS was compared with a range of RBPs based on their localization (nuclear/cytoplasmic), presence of PrLD or RNA-binding motif, and involvement in various RNA functions associated with the mRNA life cycle (splicing, translation, SG assembly…). When using TDP-43 as a bait, a significant colocalization was mainly detected with two RBPs, FUS and human antigen R (HuR) (Fig. 1*B*). The recruitment on the microtubules of the six other RBPs tested was significantly less important than that of FUS or HuR (Figs. 1*C* and S2). When the roles were reversed with FUS used as the bait, TDP-43 had the best colocalization score with FUS among all the RBPs tested as preys. Note that the colocalization score with RBPs used as preys changes depending on whether FUS or TDP-43 is used as bait. Thus, when FUS is the bait protein, G3BP1 is better localized to microtubules than HuR. Moreover, even if some of the RBPs tested as preys have rather a nuclear localization (*i.e.*, FUS, TDP-43, SAM68 (SRC associated in mitosis of 68-kDa), or U2AF65), it is still possible to detect their presence on the microtubules. Finally, no prey is detected on the microtubules in the absence of a bait, as previously reported (42). Thus, the colocalization score is systematically the highest with the TDP-43/FUS couple, revealing a strong affinity between these two RBPs among those tested. To confirm FUS and TDP-43 colocalization independently of their cytoplasmic localization, proximity ligation assays (PLA) were performed in HeLa cells (Figs. 1*D* and S3). The PLA signal indicates a higher colocalization score than previously reported (27) between TDP-43 and FUS in the nuclei of Hela cells with nearly 100% of nuclei displaying a signal. Thus, our experimental data obtained in the cellular context confirms the proteomics information from the literature (27, 28, 43) and allows us to conclude that there is a specific link between TDP-43 and FUS.

### FUS and TDP-43 mix within FUS-rich compartment

**Figure 2:**
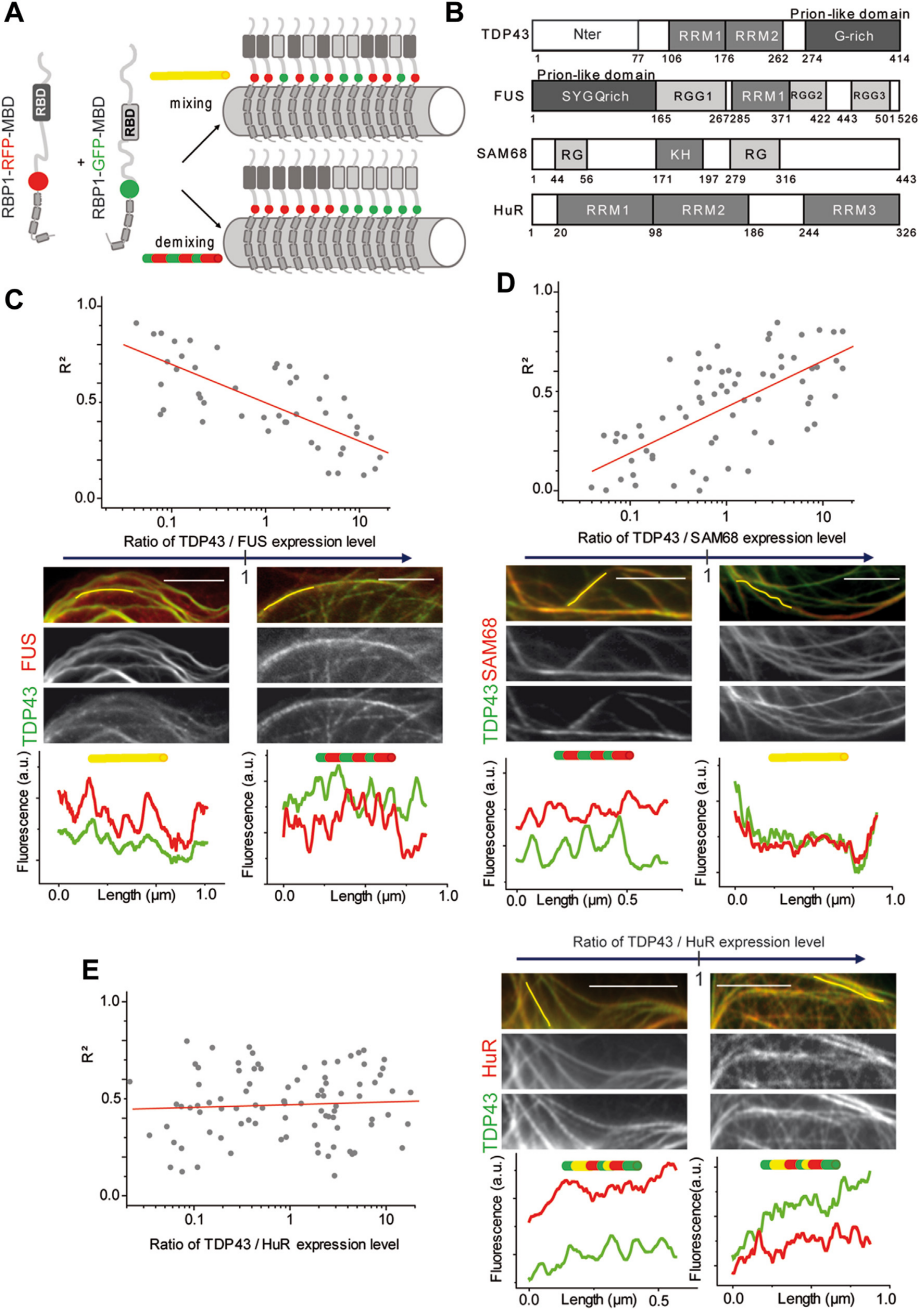
**TDP-43 is miscible in FUS-rich compartments in the cellular context.***A*, two RBPs, as indicated, are confined on the microtubule network (fused to RFP/GFP-MBD) to visualize their mixing/demixing in U2OS cells. Mixing: *yellow* microtubules. Demixing: *red* and *green* microtubules. *B*, schematic representation of domains of the RNA-binding proteins used in the microtubule bench assay to reveal their mixing according to their expression level. *C*, *upper* panel: scatter plot representing the mixing between TDP-43 and FUS, both fused to MBD according to the expression level of each construct. Each data point represents a value of determination coefficient R^2^ calculated for one cell as described in the Experimental procedures section. *Lower* panel: representative images for a low (*left* panel) and a high (*right* panel) TDP-43/FUS expression level ratio. The fluorescence intensity from the two channels along the *yellow* lines are shown below in the respective microphotographs. Scale bar represents 1 μm. *D*, same as (*C*) with the mixing between TDP-43 and SAM68 fused to MBD. *E*, same as (*C*) with the mixing between TDP-43 and HuR fused to MBD. SAM68, SRC associated in mitosis of 68-kDa; HuR, human antigen R.

The microtubule network can also be used to determine the ability of proteins to mix or demix (forming their own compartment) (39). To this end, both proteins were fused to a microtubule-binding domain. After their expression in cells, the fusion proteins were brought onto microtubules to either generate a compartment (demixing) or a homogeneous phase (mixing, Figs. 2*A* and S4) (39). In the case of RBPs, the formation of a compartment involves protein–protein interactions, in particular when they harbor low complexity domains, but also RNA–protein interactions where RNA provides a scaffold for higher protein assemblies and finally mRNA base pairing. As with polymers, the ability of macromolecules to mix depends upon their ratio (44). To analyze the formation of compartments when TDP-43 and FUS are sharing the same space, we modulated the level of expression of these proteins by changing the concentration of plasmids used during transfection. Overexpression of FUS compared to TDP-43 allows TDP-43 incorporation within FUS-rich compartments, as revealed on cell images by yellow microtubules and by an R^2^ value close to 1 (Fig. 2*C*). It corresponds to a limited occurrence of having distinct FUS- or TDP-43–rich compartments along microtubules (R^2^ is the square of the correlation coefficient, see Experimental procedures for details). Conversely, TDP-43 overexpression compared to FUS leads the microtubules to have a less uniform color with red and green clusters indicating the formation of TDP-43– or FUS-rich compartments. Then, the miscibility between the two analyzed proteins is affected resulting in a progressive decrease in R^2^ as the level of expression of TDP-43 increases. There is therefore an asymmetry in the miscibility between the two RBPs.

Next, we checked whether TDP-43 also excludes other RBPs from its compartment or whether it is specific to its association with FUS. Conversely, as FUS and TDP-43 mix within FUS-rich compartments, we examined whether this behavior is also valid for other RBPs. We selected two RBPs with different domains and aggregation propensities. SAM68 was selected as it harbors several low complexity domains (RG-rich domains) and an RNA-binding domain (KH domain) (Fig. 2*B*) with a strong propensity for multimerization or even aggregation: SAM68 is the main component of SAM68 nuclear bodies (45, 46, 47, 48). When SAM68 is brought together with TDP-43 or FUS on microtubules, the formation of distinct SAM-68–rich compartments is clearly evidenced at high expression levels of SAM68 (Figs. 2*D* and S5). The ability of SAM68 to multimerize is certainly involved in its strong propensity to form compartments on its own. Unlike SAM68, HuR does not harbor LCDs but rather multiple RRMs that can form multimers (49). Whatever the overexpression level of HuR and the nature of the other RBP partner present along microtubules (TDP-43 or FUS), R^2^ remains relatively stable and in a range of values indicating an average miscibility between HuR and TDP-43 (or FUS) (Figs. 2*E* and S5). In summary, TDP-43–rich compartments poorly recruit FUS whereas FUS and TDP-43 mix within FUS-rich compartments.

### TDP-43 and FUS form structurally different assemblies *in vitro*

**Figure 3:**
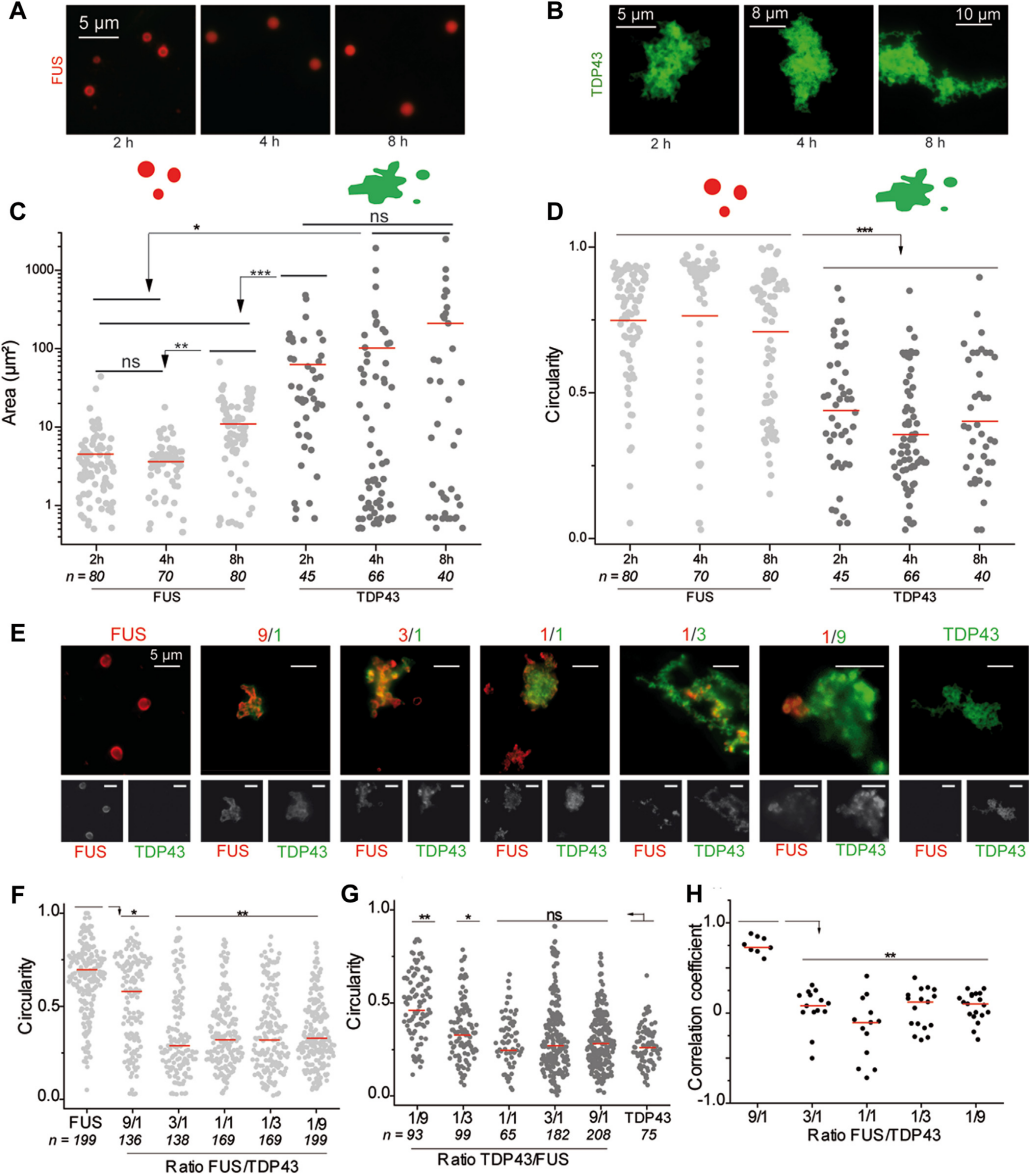
**FUS limits the segregation of TDP-43 *in vitro*.***A*, images of FUS assemblies revealed by immunofluorescence after incubation of FUS proteins at 5 μM for different incubation times. Scale bar represents 5 μm. *B*, images of TDP-43 assemblies revealed by immunofluorescence after incubation of TDP-43 proteins at 5 μM for different incubation times. *C*, scatter plot representing the area of FUS and TDP-43 assemblies during the incubation time. The plot shows the data from two independent experiments. n: number of assemblies analyzed. *Red lines* show mean values. Significances between areas of FUS and TDP-43 assemblies were obtained using *t* test; ∗*p* < 0.05; ∗∗*p* < 0.01, ∗∗∗*p* < 0.005, ns, not significant. *D*, scatter plot representing the circularity of FUS and TDP-43 assemblies along the incubation time. The plot shows the data from two independent experiments. n: number of assemblies analyzed. *Red lines* show mean values. Significances between circularity of FUS and TDP-43 assemblies were obtained using *t* test; ∗∗∗*p* < 0.005. *E*, representative images of FUS/TDP-43 assemblies after incubation of the two proteins for 2 h at different ratios ranging from 9 to 1/9. *Top*: merged signal from FUS-RFP and TDP-43 GFP. *Bottom*: images of the same assemblies but from FUS-RFP (*left*) or TDP-43-GFP (*right*). Scale bar represents 5 μm. *F*, scatter plot representing the circularity of FUS-rich phases in FUS/TDP-43 assemblies according to the FUS/TDP-43 ratio. Only the fluorescence signal from FUS-RFP was analyzed. The plot gathers the data from two independent experiments. n: number of assemblies analyzed. *Red lines* show mean values. Significances between circularity of FUS/TDP-43 assemblies were obtained using *t* test; ∗*p* < 0.05; ∗∗*p* < 0.01. *G*, scatter plot representing the circularity of TDP-43 rich phases in FUS/TDP-43 assemblies according to the FUS/TDP-43 ratio. Only the fluorescence signal from TDP-43-GFP was analyzed. The plot gathers the data from two independent experiments. n: number of assemblies analyzed. *Red lines* show mean values. Significances between circularity of FUS/TDP-43 assemblies according to the protein ratio were obtained using *t* test; ∗*p* < 0.05; ∗∗*p* < 0.01; ns, not significant. *H*, scatter plot representing the correlation coefficient between the fluorescence signals of FUS-RFP and TDP-43-GFP. Each data point represents a correlation coefficient between fluorescence intensities from *red* and *green* channels in aggregates with an area comprised between 20 to 2000 pixels. The plot shows the data from two independent experiments. Lines show mean values. Significances between correlation coefficients were obtained using *t* test; ∗∗*p* < 0.01.

The differences observed in the mixing could be linked with the architecture of both compartments. TDP-43 and FUS were then purified and incubated separately before being fixed on a glass slide and observed by fluorescence microscopy (Fig. 3, *A* and *B*). Structural characterization of higher order assemblies of TDP-43 and FUS revealed that FUS assemblies have a stable size over time (Fig. 3*C*) and a circular shape (Figs. 3*D* and S6). There is a clear structural resemblance between these assemblies and the droplets of FUS detected during the Liquid-Liquid Phase Separation process (50, 51, 52, 53, 54, 55, 56). TDP-43 assemblies are different (Fig. 3*B*). Their size increased with incubation time (Fig. 3*C*) or concentration (Fig. S6) and exhibited a filamentous appearance (Fig. 3*D* and Video SV1). TDP-43 and FUS were then co-incubated for 2 h at different ratios (Fig. 3*E*). By gradually increasing the fraction of TDP-43, we switched from structures having a morphology close to FUS to those related to TDP-43. As soon as a small fraction of TDP-43 is added, we evidence an association with the FUS droplets leading to the formation of clusters associated with a reduction in circularity (Fig. 3*F*). However, the high correlation coefficient indicates a mixing between TDP-43 and FUS in these FUS-rich clusters. When FUS is incubated with a higher fraction of TDP-43 (*i.e.*, FUS to TDP-43 M ratio of one-third and beyond), the structure of the assemblies evolves to reach the ones observed in Figure 3*B* for TDP-43 alone. The results also indicate a significant decrease in the circularity of the assemblies, independently of whether the fluorescence signal was arising from FUS or TDP-43 (Fig. 3, *F* and *G* respectively). Finally, at a FUS to TDP-43 M ratio of 3/1, the correlation coefficient collapsed and corresponded to the spatial separation between TDP-43 and FUS with the formation of TDP-43–rich or FUS-rich structures (Fig. 3*H*). Thus, FUS only seems to solubilize TDP-43 at low concentration, preventing the formation of distinct TDP-43–driven structures that occur at higher TDP-43 concentrations inside FUS-rich compartments. In the latter case, we can qualify the TDP-43–rich assemblies still interacting with the FUS compartment as subcompartments.

### RNA enhances the mixing of the two proteins *in vitro*

**Figure 4:**
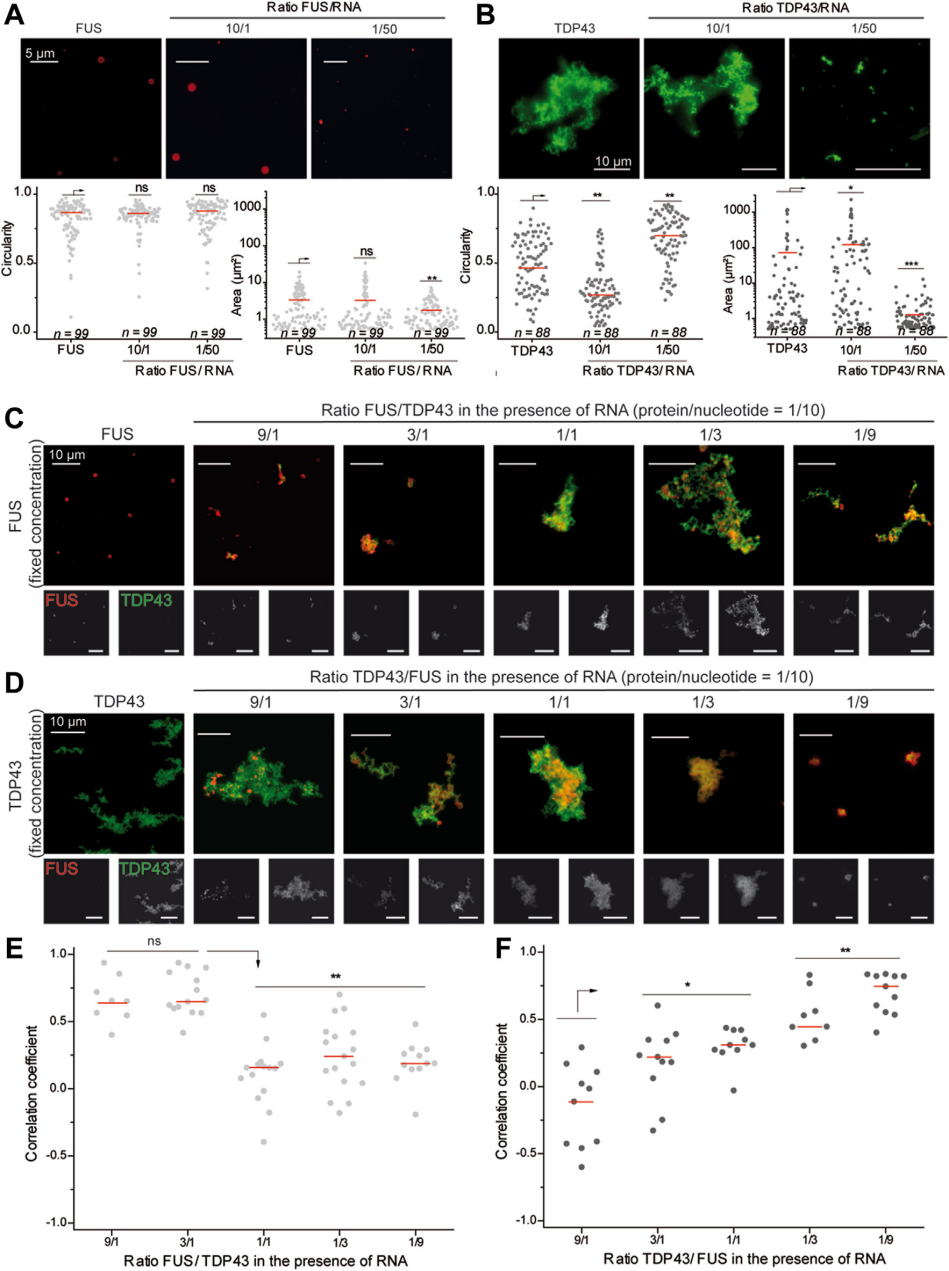
**RNA improves the miscibility of TDP-43 in FUS-rich assemblies.***A*, *Upper* panel: images of FUS assemblies revealed by immunofluorescence after incubation of FUS proteins for 2 h at 5 μM with or without 2Luc RNA. *Lower* panel: scatter plot representing the circularity (*left*) and area (*right*) of FUS assemblies according to the protein to nucleotide ratio. The plot gathers the data from two independent experiments. n: number of assemblies analyzed. Lines show mean values. Significances between circularity (or area) of FUS assemblies were obtained using *t* test; ∗∗*p* < 0.01; ns, not significant. *B*, *upper* panel: images of TDP-43 assemblies revealed by immunofluorescence after incubation of TDP-43 proteins for 2 h at 5 μM with or without 2Luc RNA. *Lower* panel: scatter plot representing the circularity (*left*) and area (*right*) of TDP-43 assemblies according to the protein to nucleotide ratio. The plot gathers the data from two independent experiments. n: number of assemblies analyzed. *Red lines* show mean values. Significances between circularity (or area) of TDP-43 assemblies were obtained using *t* test; ∗*p* < 0.05; ∗∗*p* < 0.01; ∗∗∗*p* < 0.005. *C*, images of FUS/RNA assemblies in the presence of increasing amounts of TDP-43. FUS was incubated 2 min with 2Luc RNA before TDP-43 addition and further incubation for 2 h. FUS/TDP-43 ratios were ranging from 9 to 1/9 with a concentration of FUS (5 μM) constant and a fixed protein/RNA nucleotide of 1/10. Scale bar represents 10 μm. *D*, images of TDP-43/RNA assemblies in the presence of increasing amounts of FUS. TDP-43 was incubated 2 min with 2Luc RNA before FUS addition and further incubation for 2 h. TDP-43/FUS ratios were ranging from 9 to 1/9 with a concentration of TDP-43 (1.67 μM) constant and a fixed protein/RNA nucleotide of 1/10. Scale bar represents 10 μm. *E*, scatter plot representing the correlation coefficient between the fluorescence signals of FUS and TDP-43 in the presence of 2Luc RNA under the conditions described in (*C*). Each data point represents a correlation coefficient between fluorescence intensities from *red* and *green* channels and only aggregates with an area ranging from 20 to 2000 pixels are selected. The plot shows the data from two independent experiments. *Red lines* show mean values. Significances between correlation coefficients were obtained using *t* test; ∗∗*p* < 0.01, ns, not significant. *F*, scatter plot representing the correlation coefficient between the fluorescence signals of FUS and TDP-43 in the presence of 2Luc RNA in the conditions described in (*D*). Each data point represents a correlation coefficient between fluorescence intensities from *red* and *green* channels and only aggregates with an area ranging from 20 to 2000 pixels are selected. The plot shows the data from two independent experiments. Lines show mean values. Significances between correlation coefficients were obtained using *t* test; ∗*p* < 0.05; ∗∗*p* < 0.01.

**Figure 5:**
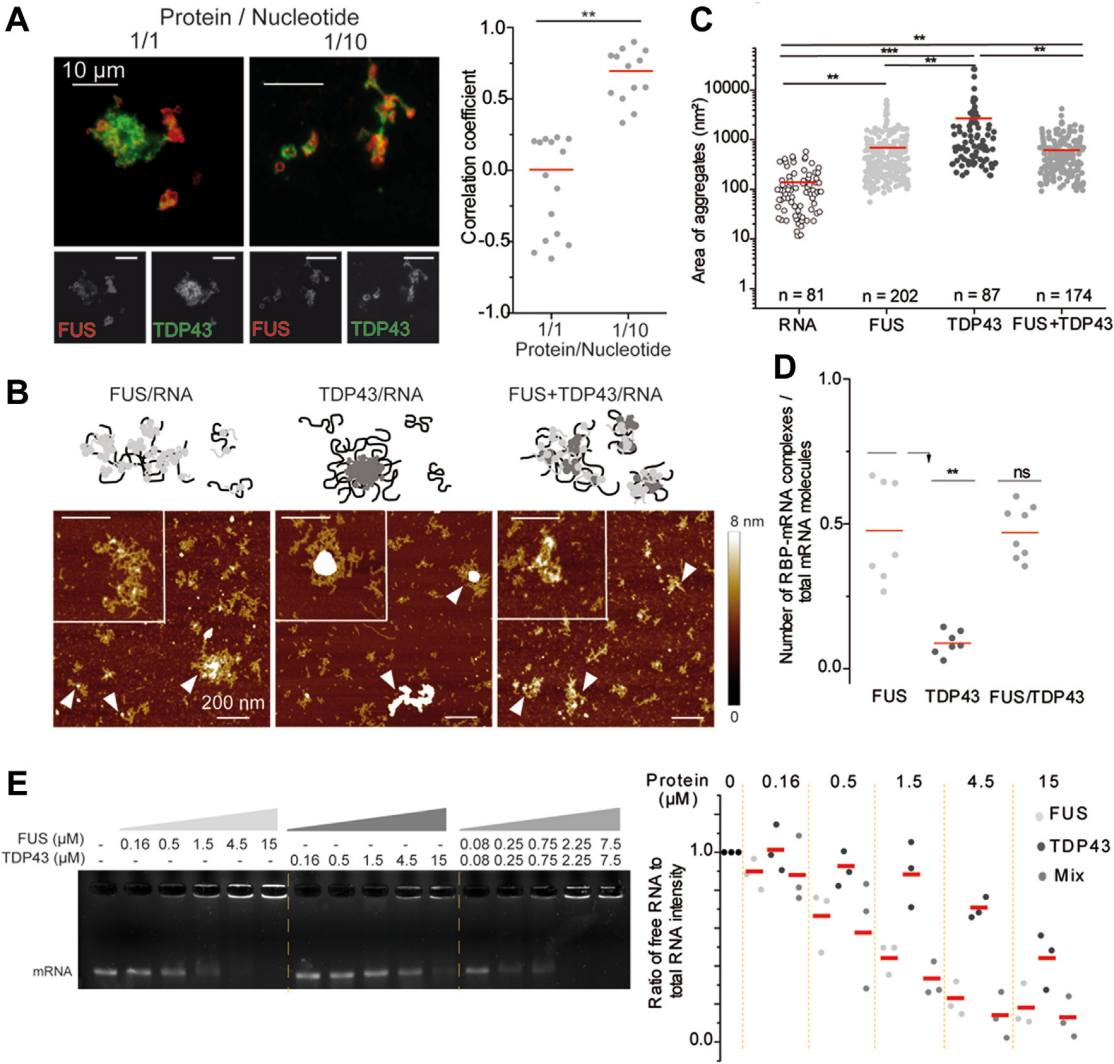
**FUS promotes the TDP-43 distribution on 2Luc RNA.***A*, *left* panel: images of FUS/TDP-43 assemblies revealed by immunofluorescence in the presence of 2Luc RNA. FUS (5 μM) and TDP-43 (1.67 μM) were incubated for 2 h with 2Luc RNA with protein/nucleotide ratio of 1/1 or 1/10. Scale bar represents 10 μm. *Right* panel: scatter plot representing the correlation coefficient between the fluorescent signals of FUS and TDP-43 according to the protein/nucleotide ratio. The plot shows the data from two independent experiments. Lines show mean values. Significances between correlation coefficients were obtained using *t* test; ∗∗*p* < 0.01. *B*, atomic force microscopy images and zooms in on specific assemblies of FUS/RNA, TDP-43/RNA, and of an equimolar mixture of the two RBPs with 2Luc RNA. Proteins (500 nM) were incubated with 2Luc RNA (protein/nucleotide ratio of 1/100) for 15 min before sample deposition and fixation. *White arrows* pointed to RNA/RBPs complexes. Z scale 8 nm. Scale bar represents 200 nm. *C*, scatter plot representing the area of RBP/RNA assemblies observed on AFM images in (*B*) and comparison with free mRNA. Only areas of RBP/RNA complexes with a height higher than 2 nm are plotted and free RNA molecules were discarded from this analysis. The plot gathers the data from two independent experiments. n: number of assemblies analyzed. Lines show mean values. Significances between areas of RBP/RNA assemblies were obtained using *t* test; ∗∗*p* < 0.01, ∗∗∗*p* < 0.005. *D*, scatter plot of the proportion of RBP/RNA complexes compared to total structures (complexes and free RNAs) adsorbed on mica surface and observed on AFM images. Each point corresponds to the same surface analyzed, here 100 μm^2^. The plot gathers the data from two independent experiments. Lines show mean values. Significances between FUS/RNA, TDP-43/RNA, and (FUS+TDP-43)/RNA samples were obtained using *t* test; ∗∗*p* < 0.01; ns, not significant. *E*, RNA mobility shift assay demonstrating the direct interaction between RBPs and RNA. One hundred fifty nanograms of 2Luc RNA were incubated with increasing concentration of FUS (for lane 2–6) or TDP-43 (for lane 7–11). For TDP-43 + FUS mixes, each protein concentration was divided per 2 to maintain the same global protein/nucleotide ratio with a 1:1 TDP-43/FUS ratio. Quantification of the free RNA band intensity of each lane (normalized, lane 1) on triplicate. AFM, atomic force microscopy.

TDP-43 and FUS assemblies are modulated by the presence of RNA. FUS assemblies remain spherical in the presence of RNA but their size decreases when the FUS/RNA molar ratio decreases (Fig. 4*A*). For TDP-43, the influence of RNA on the formation of its higher order assemblies is more visible. For a protein/RNA ratio of 10/1, RNA favors the formation of large assemblies characterized by a significant increase in the average area. At lower ratios, large TDP-43 assemblies dissociate, as expected owing to the buffering action of RNA (Fig. 4*B*) (8). Next, we probed whether the structures of TDP-43 and FUS assemblies are RNA-dependent. RNA was first incubated with FUS for few minutes and then TDP-43 was added at varying concentration until becoming dominant while the protein/RNA ratio was fixed at 1/10 (Fig. 4*C*). The resulting structures are quite similar to those obtained in Figure 3*E* in the absence of RNA (Videos SV2 and SV3). The typical pattern of FUS (spherical assemblies that associate in the form of clusters) is gradually substituted by that of TDP 43 with massive assemblies of filamentous appearance. At high TDP-43 proportion, the correlation coefficient decreases but the starting point of the demixing process is shifted towards higher proportions of TDP-43 than what was observed in the absence of RNA (Fig. 4*E*). In contrast, when TDP-43 is first incubated with RNA and then FUS is added at increasing proportions, we observed that FUS was hardly incorporated in TDP-43 assemblies as evidenced by a low correlation coefficient (Fig. 4, *D* and *F*). We then examined whether the concentration of RNA also modulate the ability of FUS and TDP-43 to mix. When the amount of RNA was increased and the molar ratio of protein to RNA nucleotide was decreased from 1 to 0.1, while keeping the FUS to TDP-43 M ratio to 3, the colocalization between the two proteins clearly increased (Fig. 5*A*).

To further explore the consequences of the mixing between FUS and TDP-43 on the structure of protein/RNA complexes, we used high resolution atomic force microscopy (AFM) to enable nanometer scale analysis of multimolecular assemblies. FUS was incubated with RNA for a few minutes to analyze the formation of RNA/FUS complexes (protein/nucleotide ratio fixed at 1/100). FUS is quite homogenously distributed among the RNAs (Figs. 5, *B* and *D* and S7) since approximately half of the RNAs adsorbed on the surface were interacting with one or more FUS proteins, leading to the formation of large assemblies weakly compacted or isolated complexes. For the same incubation time and protein/nucleotide ratio, TDP-43 repartition on RNA is less homogeneous (Figs. 5, *B* and *D* and S7). TDP-43 tends to accumulate on few RNAs, thus forming highly compacted and large structures (arrows in Figs. 5*B* and S7). When equimolar proportions of TDP-43 and FUS were incubated with RNA for the same incubation time and protein/nucleotide ratio, numerous structural changes were observed in the protein/RNA complexes. First, the proportion of RNAs complexed with proteins is close to that observed with FUS and RNA alone (Fig. 5*D*). Second, the area of RNA/protein complexes decreases considerably compared to TDP-43/RNA samples (Fig. 5*C*). In parallel, the large compact structures observed in the TDP-43/RNA samples are no longer detected. Thus, at the micrometric scale (Fig. 4, *C* and *D*), the association between TDP-43 and FUS in the presence of RNA favors the formation of large assemblies in which we noticed the presence of clusters. Therefore, at the nanometer scale, the results indicate that FUS limits the capacity of TDP-43 for self-assembly in distinct TDP-43–rich structures and promotes the formation of TDP-43 clusters embedded in FUS/RNA assemblies. The buffering of TDP-43 by FUS observed at the single molecule level was further corroborated by gel shift assays. Indeed, the combination of TDP-43 and FUS leads to their association with a slightly higher fraction of RNA than when RNA is incubated with only one of the proteins, the total RBP concentration being constant and TDP-43 alone poorly associates with mRNA (Fig. 5*E*). In addition, the presence of FUS within pre-incubated TDP-43 assemblies favors their association with RNA compared to TDP-43 and FUS assemblies considered independently (Fig. S8). Thus, the interplay between FUS and TDP-43 regulates their mutual higher order assemblies in the presence of RNA to preserve the functional binding of TDP-43 to mRNA.

### TDP-43 and FUS colocalization in SGs is mediated by the cooperative binding of TDP-43 to RNA

**Figure 6:**
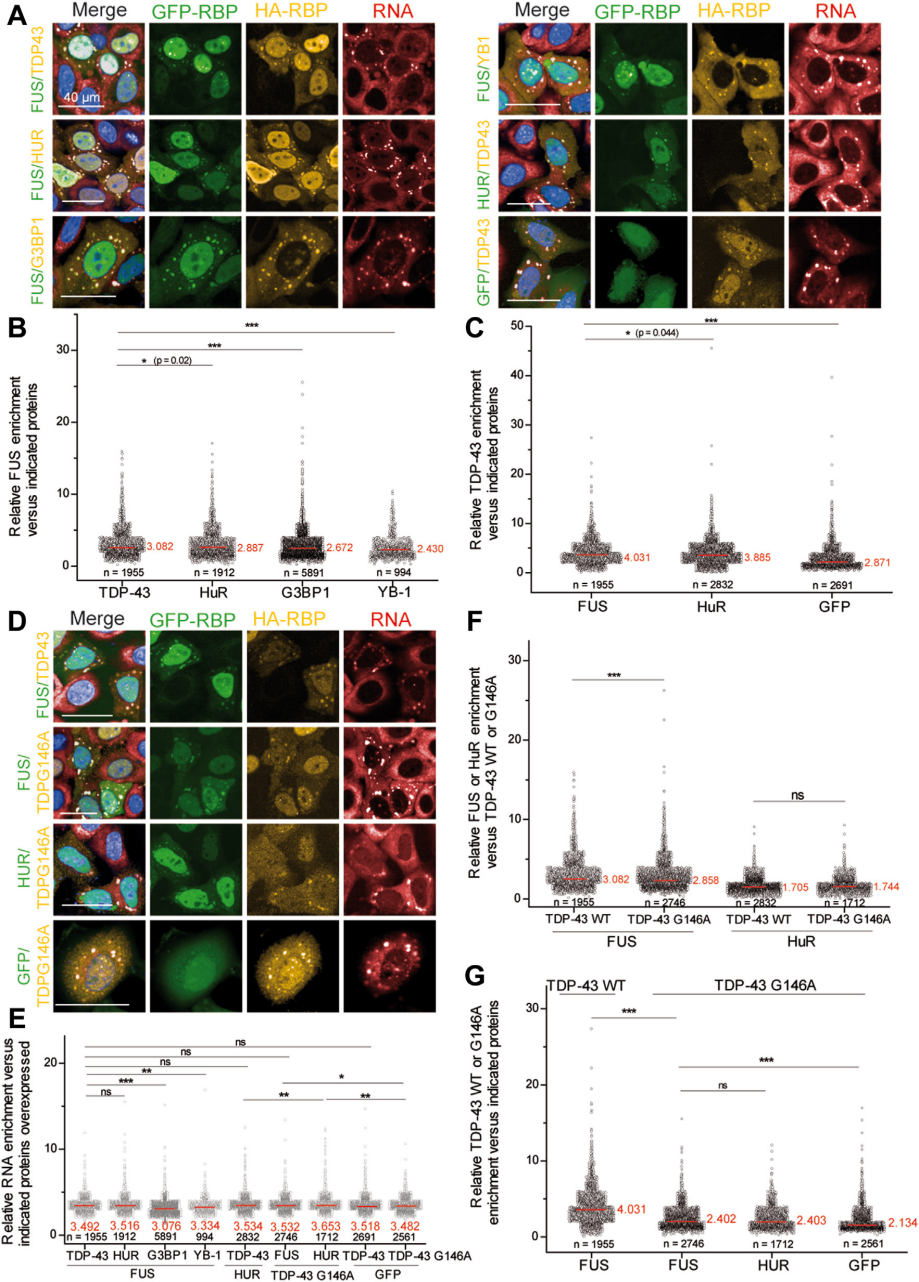
**FUS and TDP-43 colocalization in SGs is impaired by a cooperativity-defective mutation in TDP-43.***A*, representative images of HeLa cells overexpressing HA- and GFP-tagged RBP exposed to H_2_O_2_ and puromycin treatments to trigger SG assembly. Scale bar represents 40 μm. *B*, scatter plot representing the relative FUS-GFP enrichment in SGs *versus* overexpressed HA-RBPs. Significances between FUS enrichment levels were obtained using *t* test; ∗*p* < 0.05; ∗∗∗*p* < 0.005. n: number of cells analyzed; in *red* mean value. *C*, scatter plot representing the relative TDP-43-HA enrichment in SGs *versus* overexpressed GFP-RBPs or GFP alone as a control. Significances between TDP-43 enrichment levels were obtained using *t* test; ∗*p* < 0.05; ∗∗∗*p* < 0.005. n: number of cells analyzed; in *red* mean value. *D*, representative images of HeLa cells overexpressing HA- and GFP-tagged RBP exposed to H_2_0_2_ and puromycin treatments to trigger SG assembly. Scale bar represents 40 μm. *E*, scatter plot representing the relative RNA enrichment in SGs *versus* overexpressed HA and GFP-tagged RBPs. Significances between RNA enrichment levels were obtained using *t* test; ∗*p* < 0.05; ∗∗*p* < 0.01, ∗∗∗*p* < 0.005; ns, not significant. n: number of cells analyzed; in *red* mean value. *F*, scatter plot representing the relative FUS-GFP or HuR-GFP enrichment in SGs *versus* TDP-43 and TDP-43 G146A cooperative-defective mutant. Significances in FUS or HuR enrichment were obtained using *t* test; ∗∗∗*p* < 0.005. n: number of cells analyzed; in *red* mean value. *G*, scatter plot representing the relative TDP-43-HA or TDP-43 G146A-HA enrichment in SGs *versus* overexpressed GFP-RBPs or GFP alone as a control. Significances in WT and mutant TDP-43 enrichment levels were obtained using *t* test; ∗∗∗*p* < 0.005; ns, not significant. n: number of cells analyzed; in *red* mean value. HuR, human antigen R; SG, stress granule.

TDP-43 and FUS are nuclear proteins that can shuttle to the cytoplasm upon stress exposure and assemble into SGs (57, 58). Here, we overexpressed GFP-RBPs and HA-tagged RBPs in HeLa cells. Then, SG assembly was triggered by exposure to a combined puromycin/hydrogen peroxide treatment. Puromycin causes premature chain termination, which facilitates the appearance of SGs in most hydrogen peroxide–treated HeLa cells (Fig. 6*A*). Puromycin/hydrogen peroxide treatment allows to better detect cytoplasmic FUS and TDP-43 recruitment in SGs than with arsenite treatment, mostly because FUS and TDP-43 translocated in the cytoplasm after H_2_O_2_ treatment (59). Cells were imaged using an automatic HCS imager operating in confocal mode at a high resolution and we measured the RBP enrichment in SGs compared to the cytoplasm. Depending on the RBP overexpressed, the relative FUS enrichment in the SGs varies but is higher when TDP-43 is overexpressed (Fig. 6*B*), in line with the miscibility previously observed *in vitro* and on the microtubule network (Figs. 1*B* and 5B). In addition, the TDP-43 enrichment in the SGs is promoted by the overexpression of FUS compared to HuR or GFP alone (Fig. 6*C*). In order to highlight the importance of RNA binding to the ability of FUS and TDP-43 to coexist in the same compartment, we overexpressed FUS or HuR with a TDP-43 mutant (TDP-43 G146A) in which the interface between the RRMs of two adjacent TDP-43 bound to RNA is affected, resulting in an impaired cooperativity (38) (Fig. 6*D*). It appears that the average mRNA enrichment in SGs is relatively constant independently of the protein overexpressed. In particular, overexpression of either TDP-43 WT or G146A mutant with FUS does not exhibit significant differences in the mRNA enrichment in SGs (Fig. 6*E*). However, compared to TDP-43 WT, we noticed a strong decrease in the enrichment level of TDP-43 G146A in SGs independently of the co-expressed RBPs (FUS or HuR used here) (Fig. 6*G*). In addition, the enrichment of FUS but not HuR in SGs decreases when the cooperativity-defective TDP-43 mutant is overexpressed (Fig. 6*F*). This analysis in a cellular context demonstrates that TDP-43 binding to mRNA is dependent upon the interaction between FUS and TDP-43.

### FUS is not able to solubilize TDP-25

**Figure 7:**
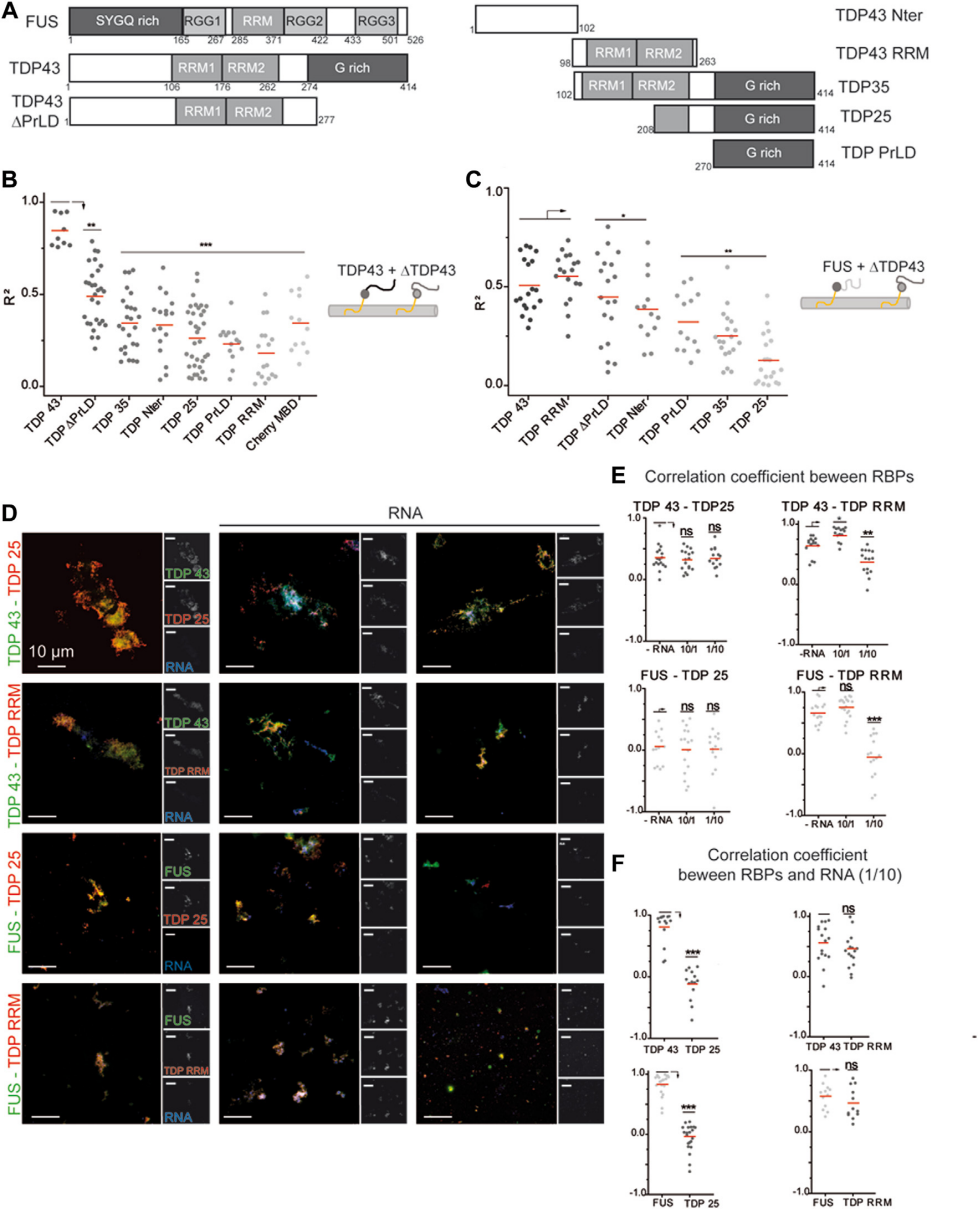
**FUS does not mix with TDP-43 truncations with an impaired RNA binding.***A*, schematic representation of the domains of TDP-43 truncations used in the microtubule bench assay to reveal their mixing with full length FUS (FL-FUS) or full length TDP-43 (FL-TDP-43). *B*, scatter plot representing the mixing between FL-TDP-43 and TDP-43 truncated forms (ΔTDP-43), both fused to MBD. Each data point represents a value of determination coefficient R^2^ calculated for one cell. Cherry-MBD construct is considered as a control as no interaction (attraction or repulsion) is expected between TDP-43 and Cherry protein. *Red lines* show mean values. Significances between determination coefficients were obtained using *t* test; ∗∗*p* < 0.01; ∗∗∗*p* < 0.005. *C*, scatter plot representing the mixing between FL-FUS full and TDP-43 truncated forms, both fused to MBD. Each data point represents a value of determination coefficient R^2^ calculated for one cell. *Red lines* show mean values. Significances between determination coefficients were obtained using *t* test; ∗*p* < 0.05; ∗∗*p* < 0.01. *D*, images of assemblies generated by the incubation for 2 h of an equimolar mix of FL-FUS (or FL-TDP-43) with TDP25 (or TDP RRM) in the absence (first column) or the presence of 2Luc RNA (*in situ* hybridization with oligo-d(T) probes). Protein to nucleotide ratio ranging from 10/1 (second column) to 1/10 (third column) with a total protein concentration of 5 μM. FL-FUS or TDP-43 were incubated less than 1 min with 2Luc RNA before the addition of the equimolar concentration of truncated forms of TDP-43. Scale bar represents 10 μm. *E*, scatter plot representing the correlation coefficient between the fluorescence signals of FL-FUS (or FL-TDP-43) and truncated TDP-43. Each data point represents a correlation coefficient between fluorescence intensities from *red* and *green* channels in assemblies with an area comprised between 20 and 2000 pixels. The plot shows the data from two independent experiments. *Red lines* show mean values. Significances between correlation coefficients were obtained using *t* test; ∗*p* < 0.05; ∗∗*p* < 0.01, ∗∗∗*p* < 0.005; ns, not significant. *F*, scatter plot representing the correlation coefficient between the fluorescence signals of proteins (FL TDP-43 or FL FUS or truncated TDP-43) and 2Luc mRNA. Each data point represents a correlation coefficient between fluorescence intensities of *red* or *green* channels and *blue* channel in aggregates with an area comprised between 20 and 2000 pixels. The plot shows the data from two independent experiments. *Red lines* show mean values. Significances between correlation coefficients were obtained using *t* test; ∗∗∗*p* < 0.005; ns, not significant. RRM, RNA-recognition motif.

FUS and TDP-43 binding to the same RNA molecules leads to higher miscibility of FUS and TDP-43 than in the absence of RNA. To determine whether RNA is the only factor controlling the miscibility of these proteins, mixing of truncated proteins was studied using the microtubule bench (Figs. 7*A* and S9). In general, the mixing of TDP-43 truncation mutants with full length TDP-43 (FL TDP-43) appears to negatively correlate with the length of the deletion (Fig. 7*B*). When FL TDP-43 interacts along microtubules with truncated FUS, the mixing with TDP-43 also decreases compared to the FL FUS (Fig. S10). None of the truncation of FUS seems to preserve the miscibility with FL TDP-43, suggesting that all the domains may contribute to the observed miscibility. If FL FUS interacts along microtubule network with truncated forms of TDP-43, we first notice that the mixing between FL FUS and TDP-43 RRMs is similar to that of FL TDP-43 (Fig. 7*C*), highlighting the importance of the TDP-43 RRMs. Consistently, the TDP-25 truncation in which the RRM1 and part of the RRM2 have been removed do not mix with FL FUS.

To correlate the results obtained in cells with those obtained *in vitro* where the RNA concentration could be controlled, the assemblies formed by incubating truncation mutants with FL TDP-43 or FUS for 2 h were observed by fluorescence optical microscopy (Fig. 7*D*). The correlation between FL proteins (TDP-43 or FUS) and TDP-25 remains low and is not modulated by the presence of RNA (Fig. 7*E*). This indicates that the proteins mix poorly with each other and the presence of RNA fails to promote their association. Moreover, RNA labeling makes it possible to detect a preferential colocalization between FL proteins and RNA (Fig. 7*F*), which seems consistent with the absence of an efficient RNA-binding domain in TDP-25. Conversely, RNA increases the mixing between FL proteins (TDP-43 or FUS) and TDP-RRM at moderate concentrations. The mixing drops at high RNA concentrations, which is certainly explained by the binding of FL TDP-43 (or FUS) and RRM to different RNAs.

**Figure 8:**
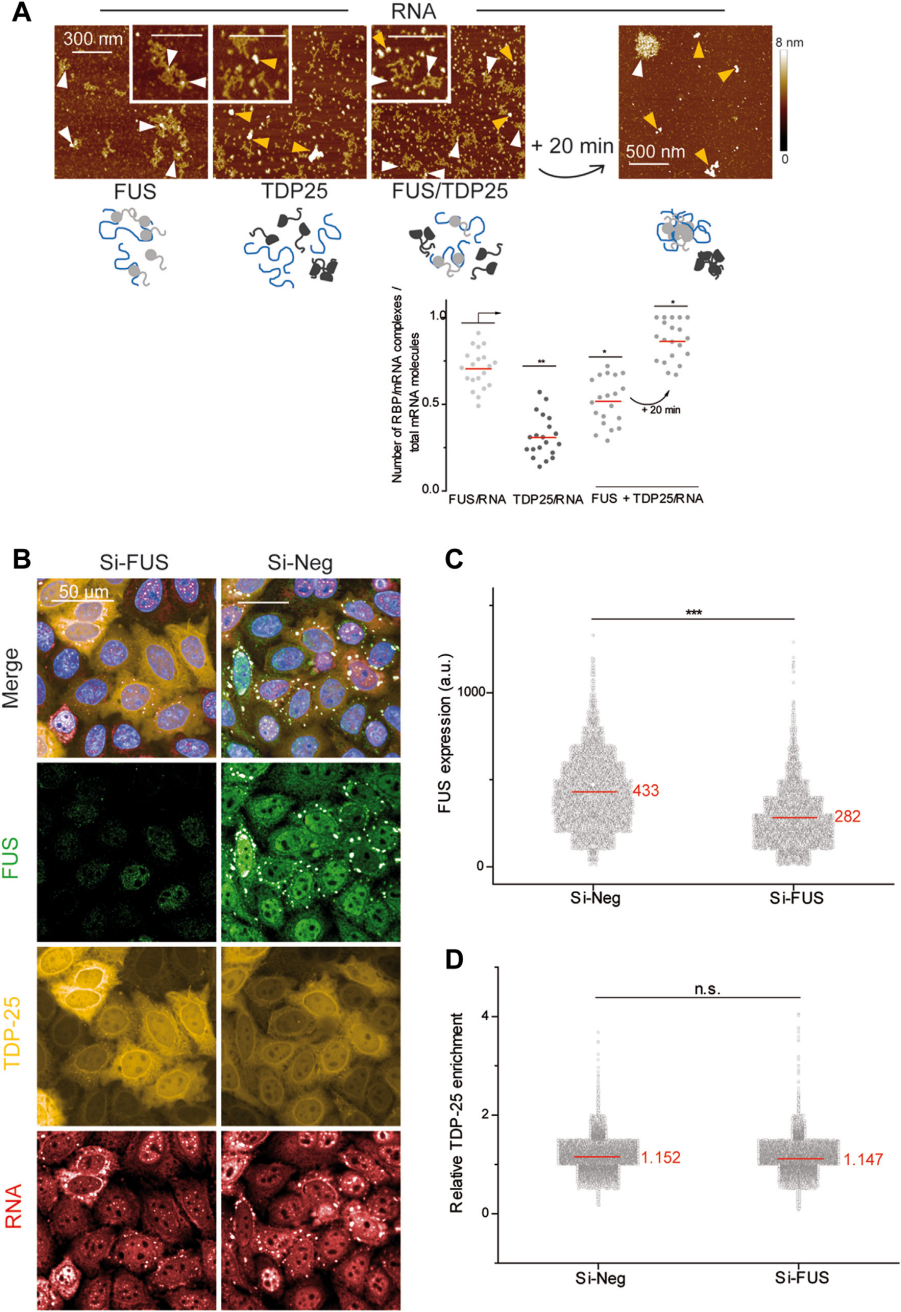
**FUS does not avoid the TDP-25 cytoplasmic aggregation.***A*, *top*: Atomic force microscopy images (and zooms in) of FUS/RNA, TDP25/RNA, and an equimolar mixture of the two RBPs with 2Luc RNA. Proteins (500 nM) were incubated with 2Luc RNA (protein/nucleotide ratio of 1/100) for 10 min (or additional 20 min for the protein mix) before sample deposition and fixation. *Orange arrows* pointed to protein aggregates apparently free of RNA; *white arrowheads* point to protein/RNA complexes. Scale bar represents 300 nm. Z scale 8 nm. *Bottom*: Scatter plot of the proportion of RBP/RNA complexes compared to total structures (complexes and free RNAs) adsorbed on mica surface and observed on AFM images. Each point corresponds to the same total surface analyzed, here 100 μm^2^. The plot gathers the data from two independent experiments. *Red lines* show mean values. Significances between FUS/RNA, TDP25/RNA, and (FUS + TDP25)/RNA samples were obtained using *t* test; ∗*p* < 0.05; ∗∗*p* < 0.01. *B*, representative images of HeLa cells overexpressing GFP-tagged TDP-25 exposed to H_2_O_2_ and puromycin treatments to trigger SG assembly with (si-FUS) or without (si-Neg) decreasing endogenous FUS levels with siRNA. Scale bar represents 50 μm. *C*, scatter plot representing FUS expression level in cells containing SG. Significance between FUS expression levels was obtained using *t* test: ∗∗∗*p* < 0.005. *D*, scatter plot representing the relative TDP-25 enrichment in SGs *versus* cytoplasmic level in cells expressing FUS (si-Neg) or not (si-FUS). Significance between TDP-25 relative enrichments was obtained using *t* test; ns, not significant. AFM, atomic force microscopy; SG, stress granule.

Taken together, our results raise the question whether lack of interactions between FUS and TDP-25 leads to the aggregation of TDP-25. This was explored through an atomic force microcopy analysis. We demonstrated previously that FUS favors the dispersion of FL TDP-43 on mRNA by using high resolution imaging (Fig. 5*B*). When FUS is incubated with RNA, FUS interacts homogenously with most of the RNAs (white arrowheads in Fig. 8*A*) before gradually gathering FUS and RNA together in the form of granules, in which the presence of RNA is still visible (as in Fig. 5*B*). TDP-25 is adsorbed on the surface as isolated proteins or compact aggregates without any RNA (orange arrowheads in Fig. 8*A*). When FUS and TDP-25 are co-incubated with RNA, some RNA–protein complexes are observed (white arrowheads) with many RNA-free proteins adsorbed on the surface. This system then evolves towards different structural assemblies, one similar to that observed for FUS/RNA complexes (white arrowheads), in contrast to the other massive and compact assemblies without apparent RNA fragments (orange arrowheads). Thus, FUS is incapable of incorporating TDP-25 within FUS/RNA complexes which could then freely form aggregates independently of the presence of RNA. We confirmed these results in cells exposed to a combined puromycin/hydrogen peroxide treatment (Fig. 8*B*). Under these conditions, FUS accumulates in SGs detected by the mRNA enrichment whereas TDP-25 is absent from these granules. After reducing the expression of FUS by siRNA (Fig. 8*C*), no significant difference in TDP-25 enrichment in SGs was detected (Fig. 8*D*). Importantly, the mRNA enrichment in the granules is independent on the FUS expression level (Fig. S11). In addition, we noticed the presence of numerous small TDP-25–rich granules as reported previously (60). These small aggregates are independent of the expression level of FUS and are not enriched in mRNA.

## Discussion

Protein aggregation is considered deleterious for cells. They must implement molecular mechanisms to prevent the aggregation of proteins, notably for RBPs that are highly prone to aggregation due to their long and self-adhesive LCDs. The presence of an elevated concentration of RNA in the nucleus is efficient to prevent nuclear RBP aggregation (61). Posttranslational modifications such as phosphorylation also interfere with the solubility of RBPs. In the case of FUS, PrLD is enriched with serine/threonine residues which have a high potential for phosphorylation. Phosphorylation of the PrLD decreases the self-adhesive FUS intermolecular interactions as reported *in vitro* (62). For TDP 43, the acetylation and phosphorylation of PrLD have been considered as factors favoring its aggregation (63, 64). However, as for FUS, phosphorylation of PrLD was recently shown to enhance the solubilization of TDP-43 (65). In addition, in the affected neurons, phosphorylation of TDP-43 may follow its aggregation (66). The exact role of TDP-43 phosphorylation in its cytoplasmic location and aggregation is thus still under debate. Finally, some proteins could act as chaperones to prevent the aggregation of RBPs. The typical example is the interplay between aggregation-prone RBPs displaying proline-rich domains and soluble proteins harboring multiple SRC homology 3 domains (48). In this case, the buffering efficiency relies on the relative SRC homology 3 and proline-rich domain concentration (67, 68).

**Figure 9:**
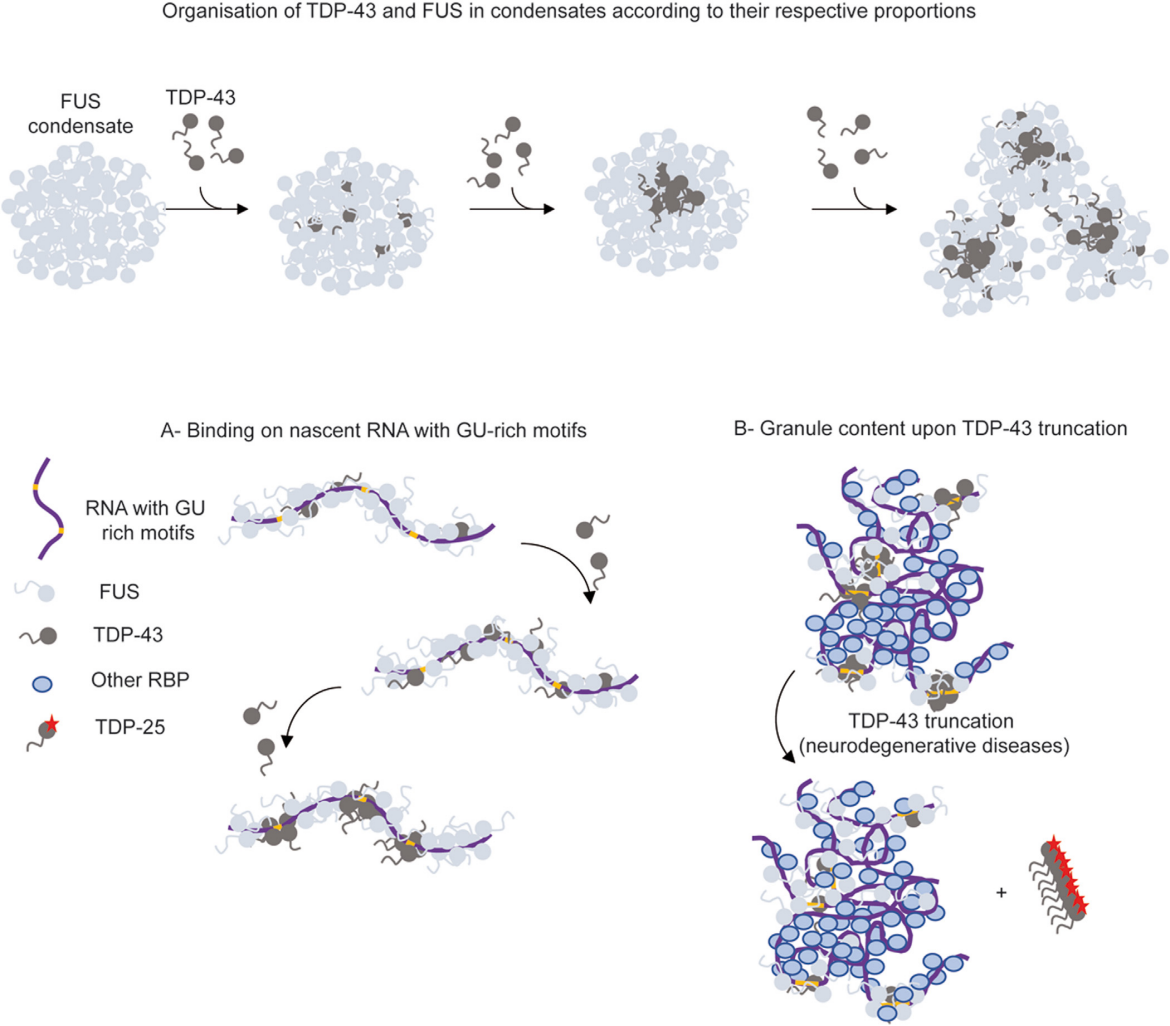
**Subcompartmentalization of TDP-43 in FUS rich-phases.***Top*: *in vitro*, without RNA, FUS assembles into condensate in which few TDP-43 molecules could be solubilized representing a typical example of subcompartmentalization. Additional TDP-43 units could integrate the FUS condensate until the solubilization limit is reached. Beyond, additional TDP-43 forms subcompartments and then promotes the coalescence of the few condensates leading to large aggregates with a filamentous appearance. The solubilization of TDP-43 into FUS rich phases may favor the accessibility of TDP-43 to nascent mRNA and its specific binding to GU-rich sequences (*bottom*, *left*). When TDP-43 loses its mRNA-binding ability (ex. truncated TDP-25), the multivalent interactions between PrLD of both proteins are not sufficient to maintain the mixing with FUS. It could promote the exclusion of TDP-25 from mRNA-rich granules like stress granules and the formation of TDP-25 insoluble aggregates (*bottom*, *right*).

Here we analyzed the consequence of the interplay between TDP-43 and FUS on TDP-43 solubility. TDP-43 is a highly aggregation-prone protein *in vitro* with a high occurrence in many neuronal cytoplasmic inclusions in neurodegenerative diseases while the occurrence of FUS-positive inclusions in neurons is significantly less important. Accordingly, FUS is comparatively more soluble in agreement with its widespread use as a model to study phase separation *in vitro*. Besides their differential tendency for aggregation, TDP-43 and FUS share a common involvement at many levels during mRNA metabolism but have not demonstrated an established overlap regarding their respective interactome. While many hnRNPs are present in the FUS interactome, TDP-43 is not considered as a major interactor (28, 29) or is absent from the FUS interactome (69, 70). For the TDP-43 interactome, the observations are also divergent as studies reported either the presence (27, 71) and absence of FUS (43). We demonstrate here that these two proteins have a strong propensity to interact in a cellular context, whether on microtubules (Fig. 1, *B* and *C*) or in SGs (Fig. 6) offering a possibility for cells to maintain a sustainable level of solubility for TDP-43. Experiments where TDP-43 was overexpressed in cells should be analyzed with caution. TDP-43 is a tightly autoregulated protein and its overexpression could result in its mislocalization and aggregation in the cytoplasm, recapitulating the main hallmark of the ALS diseases (72, 73). To determine whether FUS and TDP-43 interaction is direct or indirect, that is, involving another compound such as RNA or a common protein partner, the experiments were also carried out *in vitro*. Interestingly, the interaction between TDP-43 and FUS is reinforced by the presence of RNA, and TDP-43 preserves its functional binding to RNA in FUS:RNA assemblies (Fig. 9 top). Indeed, even if only a small fraction of TDP-43 is incorporated in FUS assemblies, it seems to be enough to obtain a more homogeneous distribution of TDP-43 on RNA (Fig. 5, *B* and *E*). TDP-43 binds specifically to GU sequences with a high cooperativity while FUS does not show a high sequence specificity. In the context of a fierce competition between nuclear RBPs for the interaction with RNAs, the crosstalk between these two RBPs could favor the access of TDP-43 to its specific sequences (Fig. 9 bottom left). Indeed, TDP-43 could be recruited in the FUS-rich phases along mRNA and gradually, *via* 2D diffusion along the mRNA, reaches its specific sites. Note that the RNA remodeling activity associated with FUS RGGs promotes the destabilization of structured RNA (34, 35), which may further increase the accessibility of TDP-43 to its specific sites. In agreement with the proposed model, FUS binds nascent RNAs, most likely in association with other FET family members (EWSR1 and TAF15) which displayed similar binding profiles on RNA (74). Interestingly, while the intron 7 of FUS pre-mRNA is known to be a target of FUS itself to orchestrate its self-regulation, a conserved TDP-43–binding site has also been identified in the same intron causing a reduction of FUS intron retention upon TDP-43 knockdown (75). This result is in line with a putative interplay between TDP-43 and FUS on introns during transcription.

FUS enhances TDP-43 solubilization; however, the reverse is less likely. Indeed, the miscibility of FUS in the TDP-43–rich phase is low (Fig. 3*H*). Even in the presence of RNA, the correlation coefficient between the signals of these proteins remains low (Fig. 4*E*), reflecting a highly limited miscibility of FUS in TDP-43–rich phase. The difference in miscibility depends upon the phase considered, which is a common behavior in polymers that have partial miscibility (76). The difficulty for FUS to mix with the TDP-43–rich phase can also be correlated to the structure of the RBP in higher-order assemblies. Indeed, while FUS is organized in small spherical aggregates with a strong resemblance to droplets, TDP-43 forms massive assemblies with a filamentous appearance (Fig. 3, *A* and *B*). By analogy with organic polymers, one can suspect a rather amorphous structure in the assemblies of FUS in which the mobility of the molecules allows the mixing with TDP-43 in connection also with the porous structure of the hydrogels formed by FUS (77) in which some RBPs like STAU1 or SMN can diffuse (52). TDP-43 assemblies would be closer to semi crystalline polymers whose structure of the dense crystalline phase prohibits any mixture with another polymer. Moreover, the mobility of molecules measured by FRAP in FUS aggregates is much higher than that measured in TDP-43 assemblies (78). In addition, this study demonstrated that FUS inclusions in cells have a diffusible component and that TDP-43 can be recruited in to these diffusible structures.

TDP-43 cytoplasmic inclusions are hallmarks of almost all cases of ALS and half of FTD cases. In contrast, FUS inclusions are less frequent in sporadic ALS and uncommon in FTD. Considering the interaction between TDP-43 and FUS and the fact that TDP-43 could be solubilized to a certain extent in the FUS-rich phases (Figs. 5*B* and 6), one may wonder whether the presence of FUS could prevent the irreversible aggregation of TDP-43 in the neurons of ALS/FTLD patients. Analysis of the composition of cytoplasmic aggregates initially produced contradictory results, some suggesting co-aggregation of TDP-43 and FUS (79, 80, 81, 82), others indicating distinct aggregation (30, 31, 83, 84, 85, 86). We demonstrated here that (i) TDP-43 and FUS colocalize in cytoplasmic SGs and (ii) overexpression of FUS favors the enrichment of TDP-43 in SGs compared to other proteins (Fig. 6). Using a defective cooperative-RNA binding TDP-43, we also showed that the enrichment of both TDP-43 and FUS in SGs is decreased compared to TDP-43 WT overexpression. However, analysis of the structure of pathologic cytoplasmic aggregates in patients with ALS suggests that these two proteins undergo independent aggregation processes (87). Interestingly, the cytoplasmic localization of FUS, independently of its aggregation, is a generic hallmark of ALS cases with TDP-43 cytoplasmic aggregation (88). Thus, these two RBPs could both be found in the cytoplasm but one in a soluble form, FUS, while the other, TDP-43, upon pathological conditions, is aggregated. The aggregation of TDP-43 could therefore be linked to the disruption of its interaction with FUS on their common mRNA targets, either due to mutations or truncations (Fig. 9 bottom right). Here we demonstrate that when TDP-43 loses its RNA-binding capacity as in the case of TDP-25 truncation, it is no longer in the environment of FUS, which promotes TDP-25 aggregation. TDP-25 is widely represented in cytoplasmic aggregates in the brain of both FTLD and ALS patients (89, 90, 91) and the loss of functional TDP-43 and FUS interplay could participate in the cytoplasmic aggregation of TDP-25.

From the data at different scales, *in vitro* and in cells presented herein, we have demonstrated that the interplay between TDP-43 and FUS allows TDP-43 to be recruited in FUS-rich phases. TDP-43 recruitment preserves its interaction with RNA and enables the formation of TDP-43–rich subcompartments. This finding is of importance since nuclear TDP-43 oligomerization is required for alternative splicing of numerous RNA targets (92, 93) and for preserving its nuclear localization (94). This relationship opens also news perspectives to better understand the mechanisms by which TDP-43 forms aggregates in a pathological context.

## Experimental procedures

### Cell experiments

#### Plasmid preparation for MT bench bait/prey method

For the microtubule bench method, plasmids encoding proteins of interest fused to RFP and microtubule-binding domain of Tau (MBD) were produced as previously described (39, 41) and are summarized in Table S1. cDNAs of full length FUS and TDP-43 were amplified using primers containing Pac1 and Asc1 restriction sites and the resulting fragments were inserted into the RFP-MBD-pCR8/GW/TOPO plasmid predigested with the corresponding restriction enzymes. Then recombination was done (Gateway LR Clonase II Enzyme mix, Invitrogen, cat n°11791020) in the mammalian expression vector pEF-Dest51 (Invitrogen). GFP-fused constructs were obtained as described previously (41). cDNAs encoding for genes of interest were amplified and inserted into pEGFP-N1 vector (Clontech). Final constructs were checked by conventional Sanger sequencing.

#### Cell culture, transfection, and fixation for MT bench experiments

U2OS cells were used for the MT bench given their well-suited morphology to visualize microtubules. They were grown in Dulbecco’s Modified Eagle’s Medium (high glucose, Sigma) with 10% FBS and penicillin/streptomycin 100 μg/ml (GIBCO Life Technologies). When confluent, cells were transferred into 4-well dishes on 12 mm coverslips for transfection. U2OS cells were transfected with 500 ng (1:1 ratio) of plasmids RBP1-RFP-MBD/RBP2-GFP (FUS-RFP-MBD and TDP-RFP-MBD with HuR-GFP, G3BP-GFP, YB1-GFP, Lin28-GFP, LARP6-GFP, SAM68-GFP, and U2AF-GFP) with lipofectamine 2000 (Invitrogen) added to the culture medium. Medium was changed after 4 h incubation to remove lipofectamine, followed by overnight incubation at 37 °C under a controlled atmosphere (5% CO_2_). After washing with PBS, cells were fixed with methanol 100% at −20 °C for 20 min and with paraformaldehyde (PFA) 4% in PBS for 30 min at 37 °C. Finally, the cells were mounted on glass slides with MOWIOL (Sigma).

#### Observation and measurement of the correlation coefficient in the bait/prey method

Images of transfected cells were acquired with an oil-immersed objective 63×/1.4 on an inverted microscope (Axiovert 220; Carl Zeiss 5 MicroImaging, Inc, Hamamatsu C10600, Axio Vision software). Exposure times were adjusted to obtain comparable fluorescence intensity between the different channels. Images were cropped for each cell and intensity and contrast parameters were adjusted with ImageJ software. Correlation between RFP and GFP signals were determined as previously described (48) and process is summarized in the Supplemental file S1. Briefly, images were treated with ImageJ software and intensity parameters were adjusted to highlight microtubules. A line crossing several microtubules was drawn (10–20 μm length) tangent and close to the nucleus and fluorescence intensities along this line were measured for both colors. A plot was generated with peaks corresponding to microtubules. If the proteins of interest interacted on microtubules, overlapping of peaks for green and red signals were observed. The line profiles for each channel were transformed into numerical values. A Pearson correlation coefficient was calculated using Microsoft Excel CORREL function based on these values. For each condition, three lines per cell and more than ten cells were analyzed.

#### Plasmid preparation for MT bench compartmentalization method

HuR-RFP-MBD, Sam68-RFP-MBD, FUS-GFP-MBD, and TDP-43-GFP-MBD plasmids (Table S2) were obtained as described previously for bait/prey method (39). cDNA for truncated forms of TDP-43 and FUS (Table S2) were amplified using designed primers containing Pac1 and Asc1 restriction sites. Resulting fragments were inserted into RFP-MBD-pCR8/GW/TOPO or GFP-MBD-pCR8/GW/TOPO plasmids previously digested with the corresponding restriction enzymes. Then recombination step was done (Gateway LR Clonase II Enzyme mix, INVITROGEN, cat n°11791020) in the mammalian expression vector pEF-Dest51 (Invitrogen) and the correct clones were confirmed by DNA sequencing.

#### Cell transfection and preparation for MT bench compartmentalization method

U2OS cells were prepared, transfected, and fixed as described previously. To study the compartmentalization of proteins according to their concentration, cells were transfected with FUS-RFP-MBD/TDP-43-GFP-MBD, Sam68-RFP-MBD/FUS-GFP-MBD, Sam68-RFP-MBD/TDP-43-GFP-MBD, HuR-RFP-MBD/TDP-43-GFP-MBD, or HuR-RFP-MBD/FUS-GFP-MBD plasmids at varying ratios (0.5/5, 0.5/2.5, 0.5/1.25, 1/1, 2.5/1, 5/1, 10/1 μg/μg of plasmids). To avoid any bias linked to differences in fluorescence intensities during the analyses, acquisition parameters (exposure time and exposition intensity) were adjusted to obtain comparable levels for green and red intensities. RBP1/RBP2 expression level ratios for each plasmid ratio were recalculated using adjusted parameters as follows: exposure time [Expo] and measured fluorescence intensity [I] for each cell were divided by the mean values of exposure time and fluorescence intensities respectively for at least 20 cells. [plasmid] corresponds to the plasmid ratio (in μg/μg) used for the transfection and is ranging from 1/10 to 10.

$$\frac{RBP1\;-\;GFP}{RBP2-RFP}expression\;level\;=\;\frac{\left(Igreen/Ired\right)}{\left(Igreen/Ired\right)mean}\;x\;\frac{\left(\frac{Expogreen}{Expored}\right)}{\left(\frac{Expogreen}{Expored}\right)mean}\;x\;\frac{Plasmid\left(RBP1\;-\;GFP\;-\;MBD\right)}{Plasmid\left(RBP2\;-\;RFP\;-\;MBD\right)}$$

For truncated forms, cells were transfected with a 1:1 ratio.

#### Analysis of images, compartmentalization detection, and measurement of determination coefficient

Images were cropped for each cell and intensity and contrast parameters were adjusted with ImageJ software. For each condition, the resulting images were analyzed with CellProfiler software to measure the determination coefficient (described in Fig. S4). Briefly, algorithms detect cells based on their nuclei and then, tubular structures (5–50 pixels) over a threshold based on their fluorescence intensities. Upper quartile fluorescence intensities for green and red colors were extracted within the selected structures. Generated data were filtered based on their eccentricity (>0.9) and background was removed. For each cell, a determination coefficient R^2^ was estimated by linear regression between green and red fluorescence intensities from at least 100 values per cell (Microsoft Excel).

#### PLA: proximity ligation assay

Duolink PLA technology kit (Sigma Aldrich) was used according to the manufacturer’s protocol. HeLa cells were prepared in 96-well plates at a density of 20, 000 cells per well. Cells were fixed with PFA 4% for 20 min at 37 °C and then, incubated in blocking buffer (3% BSA, 0.1% Triton X-100 in PBS) for 1 h at 37 °C. Primary antibodies for FUS (α-FUS rabbit, mAB ABnova) and TDP-43 (α-TDP-43 mousse, pAB Novus Bio) were diluted to 1:1000 in blocking buffer and incubated for 90 min at room temperature in blocking buffer in a humidity chamber. Four replicates from two independent experiments were done for this condition. U2AF antibodies (Fig. 1*D*) have been used for control (U2AF65 rabbit polyclonal Ab, A303-667A, Bethyl, and U2AF65 mouse monoclonal Ab, clone MC3). In parallel, two negative controls were done without primary antibodies or only FUS antibody (Fig. S3). PLA probes (Minus and Plus, α-rb, α-ms) were diluted 1:5 in the appropriate buffer from the kit and then incubated for 1 h at 37 °C with different samples. Cells were washed several times with the washing buffer (10 mM Tris, 150 mM NaCl, 0.05% Tween) before ligation step with ligase (5× diluted ligation stock and 40× diluted ligase) for 30 min at 37 °C. After washing with PBS, amplification was done using 50× diluted amplification stock and 80× diluted polymerase for 100 min at 37 °C. Cells were finally washed with 1×, then 0.1× Tris buffer (200 mM Tris, 100 mM NaCl). DAPI was used to stain nuclei. Samples were stored in PBS without mounting at 4 °C. Acquisitions were done using Opera Phenix Plus confocal microscope with 20× magnification in air using Harmony software. Data were treated with ImageJ and cellProfiler softwares. Each point plotted on the graph represents the percentage of cells with at least one PLA signal (red dot) in the nucleus among 30 cells analyzed.

#### Stress granule experiments

Stress granules experiments were performed as previously described (38, 59). HeLa cells were cultured in Dulbecco’s modified Eagle’s medium supplemented with 10% FBS in the presence of penicillin and streptomycin (100 μg/ml) (GIBCO Life Technologies). Cell cultures were maintained at 37 °C and 5% of CO_2_ in an incubator. HeLa Cells were plated in 96-well plate (PerkinElmer). The plasmids were designed to express, in mammalian cells, FUS and HUR proteins with a GFP tag and TDP-43 WT or mutants, FUS, HUR, G3BP1, and YB1 bearing an HA tag peptide on N terminus. For transfection, cells were incubated in the presence of 0.3 μg of the plasmid followed by the addition of lipofectamine 2000 reagent (0.2 μl/sample).

##### Oxidative stress

HeLa cells were treated with puromycin (2.5 μg/ml) and hydrogen peroxide (H_2_O_2_) (300 μM) for 90 min at 37 °C in a CO_2_ controlled chamber. After treatment, cells were washed twice with warm-PBS and fixed with 4% PFA diluted in PBS for 20 min at 37 °C. Cells were then incubated with 70% ethanol for 10 min at room temperature followed by incubation in the presence of 1 M Tris–HCl pH 8.0 for 5 min.

##### *In situ* hybridization

To visualize mRNA, HeLa cells were incubated with a poly-dT oligonucleotide coupled with Cy-2 (Molecular Probes Life Tech.) for 1 h at 37 °C. Washings were carried out using 4× and then 2× SSC buffer (1.75% NaCl and 0.88% sodium citrate, pH 7.0). To visualize HA-tagged proteins, cells were incubated overnight at 4 °C with an anti-HA mouse, primary antibody (Sigma-Aldrich) diluted (10^3^) in blocking buffer containing 0.1% Triton X-100. After washings with PBS, cells were incubated with a secondary goat anti-rabbit IgG antibody (10^3^ coupled to Alexa Fluor Plus 594 (Molecular Probes Life Tech.)) for 60 min at room temperature. For nuclei visualization, cells were incubated for 1 min with DAPI (0.66 mg/ml) (Sigma-Aldrich).

Quantifications were performed with Opera Phenix Plus High Content Screening System (PerkinElmer) in the confocal mode. The Harmony v4.8 software was used to automatically detect the SGs in cells. Overall cytoplasmic expression of proteins was measured along with their signal intensities in SGs; their ratio gives an enrichment in SGs.

#### RNA interference

To silence FUS, HeLa cells were transfected with 0.15 μg/well of siRNA duplexes using lipofectamine 2000. A non targeting siRNA (AllStars Negative Control QIAGEN Cat # 10272281) served as a negative control. Cells were first transfected with the siRNA and incubated at 37 °C for 24 h and then transfected with TDP-25-RFP vector plasmid (0.25 μg/well) and incubated for 24 h at 37 °C. Cells were then fixed, incubated with a poly-dT oligonucleotide coupled with Cy-2 and a murine anti-FUS primary antibody (Sigma Aldrich) as previously described.

### *In vitro* experiments

#### *In vitro* RNA transcription

RNA was produced by *in vitro* transcription as previously described (48). Briefly, linearized plasmid pSP72-2Luc, containing two full-length cDNAs encoding *Renilla reinformis* and *Photinus pyralis* luciferases, served as a template for 2Luc mRNA (∼3000 nt). HiScribe T7 High Yield RNA Synthesis Kit (NEB) was used for *in vitro* transcription, according to the manufacturer’s protocol. Synthesized RNA was purified using phenol/chloroform extraction.

#### Plasmid preparation for *in vitro* production of protein

To generate recombinant proteins, FL and truncated TDP-43 (TDP-25 and TDP-RRM) and FUS expression vectors were produced using specific primers (Table S3). pMS2-derived intermediary plasmids were used to add an HA tag to the coding sequences. Fragments were ligated between Nhe1 and BamH1 restriction sites of the plasmid. A second cloning was done to recover the sequence of interest with the HA tag. Finally, the sequences containing the HA tag were transferred in a bacterial expression vector pET22b (Novagen) between Xho1 and HindIII restriction sites. pET22b contains a His_6_ tag for purification, a Lac sequence for IPTG-induced protein synthesis, and confers ampicillin resistance.

#### Production and purification of proteins

N-terminal His6-tagged recombinant proteins (FL FUS, FL TDP-43, and truncated forms) were expressed in *Escherichia coli* BL21 (DE3) strain. After transformation (heat shock 42 °C), single colonies grown on LB-agar medium were transferred to 100 ml 2 YT medium containing ampicillin and the culture was incubated overnight at 37 °C under stirring. The total volume of the preculture was then transferred in 1 l culture medium and grown until the optical density reached 0.6. Gene expression was induced by adding 1 mM IPTG and bacteria were grown further for 4 h at 37 °C before being collected by centrifugation. The pellet was resuspended in the Tris buffer containing urea (25 mM Tris, 8 M urea, 2 M NaCl, 5 mM imidazole, 0.5 mM DTT, pH 7.4) with PMSF (Sigma) and protease inhibitors (*EDTA-free protease inhibitor cocktail Roche*). Urea was used to keep proteins soluble as they are highly prone to aggregation. Bacterial cells were lysed by sonication (Bioblock Vibracell sonicar, model 72412). Supernatant was finally recovered by ultracentrifugation (75 000*g*, 40 min, 4 °C).

For purification, supernatant was incubated with Ni-Nta-agarose beads (Qiagen) for at least 2 h at 4 °C and then loaded on a column equilibrated with Tris-Urea buffer. Following several washes with a gradient of imidazole (between 10 mM to 100 mM), the purified proteins were eluted with 250 mM, 500 mM, and 1 M imidazole. All fractions (washes and elutions) were analyzed by 12% SDS-PAGE to identify fractions containing purified proteins. Identified fractions were pulled and loaded on desalting column (PD10 Sephadex G-25M, GE-Healthcare) equilibrated with urea buffer (Urea 8 M, Tris 25 mM, NaCl 200 mM, DTT 0.5 mM, pH 7.5) to remove imidazole. Finally, protein samples were concentrated by centrifugation (SpinX^R^ Concentrator 5KMCO or 10KMWCO, Corning). Samples were stored at −80 °C till use.

#### Electrophoretic mobility shift assay

2Luc mRNA was heated to 80 °C for 1 min and then cooled at room temperature and diluted to obtain 150 ng/well. For Figure 5*E*, RNA was mixed with proteins in a Hepes buffer (20 mM Hepes, 25 mM KCl, 2 mM MgCl_2_, 1 mM DTT, pH 7.4) and incubated for 15 min at room temperature. For Fig. S7, proteins were pre-incubated for 50 min in Hepes buffer and then 2Luc mRNA was added (115 ng/well) for 10 min. Samples were analyzed on 1% agarose gel (with BET 5%, in TAE 1×) for 1 h at 25 V.

#### Immunofluorescence observation of RBP assemblies *in vitro*

Proteins were diluted and mixed (with or without 2Luc RNA) in a Tris buffer (Tris 20 mM, KCl 20 mM, MgCl_2_ 2 mM, DTT 1 mM, pH 7.5) and incubated 2 h at 37 °C before fixation. Glass slides were pretreated with poly-lysine 0.2 mg/ml for 15 min and then with glutaraldehyde (2% in water) for 40 min. Following different incubation times, protein samples were deposited on the slides. After 5 min, glutaraldehyde 2% was added for 15 min to fix samples. Slides were washed with PBS, then NaBH_4_ (1 mg/ml, freshly prepared solution) was added to bleach glutaraldehyde fluorescence. For immuno-marking, specific primary antibodies against proteins or tags (Table S4) were used, after a blocking step (BSA 1%, Triton 0.25% in PBS). During the blocking step, sample could also be incubated for 1 h with oligo-dT-dig probe (1:1000, polyT-dig Sigma HA09131354-004). Antibodies were diluted 1:500 in blocking buffer and incubated for 2 h at 37 °C in humidity chamber. Secondary antibodies were then used to stain the samples. After 2 h incubation, slides were washed with PBS, mounted (DAKO^R^ Fluorescent Mounting Medium S3023), and stored at 4 °C. Observations were done with an optical fluorescence microscope (Leica Microsystems DM4B, Hamamatsu C11440 Digital Camera) with an oil immersed objective with 1.6 × 63x magnification and acquisitions were made with Las X software (2.0.0.14332). Images were treated with ImageJ software. Some acquisitions were obtained using a confocal microscope (TCS SP8, Leica), with imaging platform ImCy (Généthon).

#### Determination of correlation between signals in aggregates

Correlation between fluorescent signals was determined by using an algorithm developed with CellProfiler software. Briefly, the program detects aggregates with an area ranging from 20 to 2000 pixels based on the fluorescence contrast with background (Threshold strategy Global with Ostu method) for green and red channels. A layer is defined for each object to detect clusters (of 2–150 pixels) in these aggregates for the two channels. This step is equivalent to forming a grid on aggregates in which fluorescence intensity is measured (MeasureObjectIntensity function on CellProfiler software). The generated data are then exported and used to calculate a correlation coefficient (Pearson, with CORREL function of Microsoft Excel software), reflecting the distribution of green and red fluorescence signals in aggregates. This coefficient is comprised between −1 and 1: signals are distinct under 0, heterogeneous around 0, and well mixed close to 1. About 8 to 12 images were analyzed for each condition tested. Area or circularity of aggregates were obtained using MeasureObjectSizeShape function on CellProfiler software. Significance between correlation coefficients were obtained using *t* test; ∗*p* < 0.05; ∗∗*p* < 0.01; ∗∗∗*p* < 0.005; ns, not significant.

#### AFM imaging

The observation of protein/RNA complexes using AFM was performed as described previously (40). Full length or truncated proteins were incubated at room temperature alone, mixed or with RNA in a Tris buffer (10 mM Tris, 15 mM KCl, 2 mM MgCl_2_, 1 mM DTT, 10 mM Putrescine, pH 6.8). A 10 μl droplet was deposited on a freshly cleaved mica surface which was quickly immersed in a diluted uranyl acetate solution (0.02% in water) to fix the sample. The samples were dried with filter paper before imaging. AFM scans were obtained using PeakForce tapping mode in air with Nanoscope V Multimode 8 software (Bruker). This model enables continuous force-distance curves recording using Scanasyst-Air probes (Bruker). Images were captured at 1512 × 1512 pixels at a line rate of 1.5 Hz. The “particle analysis” tool on the Nanoscope Analysis software (version 1.70) was used to determine the molecular dimensions of particles (proteins aggregates and mRNA:protein complexes) from at least two independent samples. Basically, a threshold of 2 nm was applied to discard small particles or patterns (uranyl acetate background) and free mRNA from the analysis (Fig. S7). The proportion of RBP/RNA complexes is estimated from at least six scanned areas (total area = 100 μm^2^) and ± SD represents the discrepancy between each scanned areas. Significance of areas and ratios were obtained using *t* test; ∗*p* < 0.05; ∗∗*p* < 0.01; ∗∗∗*p* < 0.005; ns, not significant.

## Data availability

All the data presented in this study are available upon request from the corresponding author.

## Supporting information

This article contains supporting information (39, 41, 48).

## Conflicts of interest

The authors declare that they have no conflicts of interests with the contents of this article.

## Supporting information

Supporting information

Video SV1

Video SV2

Video SV3
