# Supplementary material for: RNA and the RNA-binding protein FUS act in concert to prevent TDP-43 spatial segregation
Source: J Biol Chem. 2024 Feb 2;300(3):105716. doi: 10.1016/j.jbc.2024.105716 (PMC10912363; doi:10.1016/j.jbc.2024.105716)
Supplement: Supporting information [file mmc1.pdf]

Supplementary figure legends

Figure S1:

Schematic representation of the correlation coefficient calculation procedure. In brief, images of the bait and the prey were merged into a single red-green image. A line crossing apparent microtubules was added and fluorescence intensity profiles along the line were generated for each channel. The line profiles were transformed into numerical values by obtaining a list of the fluorescence intensities along the line. The acquired lists were used to calculate the correlation coefficient between fluorescence intensities from both channels. At least three lines and more than ten cells from two independent experiments were analyzed for each bait-prey couple and used in a subsequent statistics analysis.

Figure S2

Example of U2OS cells co-transfected with the constructions encoding TDP-43 (left handed panel) or FUS (right handed panel) fused to RFP-MBD and the plasmid expressing different RBPs fused to GFP. Scale bar: 10 µm.

Figure S3

Upper panel: Representative images for proximity ligation assay (PLA) revealing the co-localization of TDP-43 and FUS in HeLa cells. Cells were fixed, incubated with anti-FUS and anti-TDP-43 antibodies and incubated with oligonucleotides probes, ligase and polymerase as described in the Materials and Methods section. Scale bar: 150 µm. Lower panel: scatter plot representing the ratio between the area of the PLA signal in the nucleus and the area of the nucleus. n: number of nuclei analyzed. Red lines show mean values. For each conditions, the percentage of cells with a PLA signal is indicating. ***, $p < 0.005$; t-test.

Figure S4

Schematic representation of the determination coefficient (R^2) procedure calculation using the MT bench assay when two RBPs are brought on the MTs.

Figure S5

Left handed panel: scatter plot representing the mixing between FUS and HuR, both fused to MBD according to the expression level of each construct. Each data point represents a value of determination coefficient R^2 calculated for one cell as described in the Materials and Methods section and supplementary figure S4. Representative images for a low (left handed panel) and a high (right handed panel) FUS/HuR expression level ratio.

Right handed panel: same as above but for the mixing between FUS and SAM68.

Figure S6

Top: Images of FUS assemblies revealed by immunofluorescence after incubation of FUS protein at different concentrations for 2 hours. Scale bar: 40 µm. Scatter plot representing the circularity of FUS assemblies for different concentrations. The plot shows the data from two independent experiments. n: number of assemblies analyzed. Red lines show mean values. Significances between circularity of FUS assemblies were obtained using t test; *, $p < 0.05$; **, $p < 0.01$.

Bottom: the same but for TDP-43 assemblies.

Figure S7

Process for the determination of the proportion of RBP/RNA complexes and their area from AFM image analyzes. Scale bar: 10 µm. Top panel: in this example, 22 RNA molecules were identified on the surface but only 2 have a maximum height above 2 nm (ratio of 0.09). Areas of each molecule portion detected after applying this threshold of 2 nm were determined using the particle analysis tool of the Nanoscope Analysis software (version 1.70) from Bruker. Middle panel: on the same image as Figure 5B, among the 27 molecules or complexes detected, 18 (blue arrowheads) are considered as RNA/protein complexes since their maximum height is above the threshold value of 2 nm. Areas of the complex detected were plotted. Bottom panel: when TDP43 is mixed with RNA, the proportion of complexes is lower than with FUS (0.17 and 0.67 respectively) but the areas of the complex portion with a height higher than 2 nm (blue arrowheads) increase.

Figure S8

RNA mobility shift assay duplicate of TDP-43 and FUS interacting together or independently with RNA. 115 ng of 2Luc RNA were mixed for 10 min with preincubated (50 min) TDP-43 (fixed at 3 µM) or FUS (concentration ranging between 0.1 to 9 µM) or a mix of both. Amounts of RNA interacting with RBP were quantified for each lane and compared for the mix (lane 8-12, grey circles) with the sum of lane 2 plus lane 3 to 7 (grey diamonds). Arrows highlight conditions were the amount of RNA interacting with RBP is higher when RBPs are mixed than considered independently

Figure S9

Representative images of U2OS cells co transfected with different TDP-43 constructs and FUS FL revealing diverse degrees of mixing in comparison with cell transfected with RFP and GFP-TDP-43-MBD. Scale bar: 15 µm.

Figure S10

Top: schematic representation of the domains of FUS truncations used in the microtubule bench assay to reveal their mixing with full length TDP-43. Bottom: scatter plot representing the mixing between TDP-43 full length and FUS truncated forms, both fused to MBD. Each data point represents a value of determination coefficient R^2 calculated for one cell. Red lines show mean values. Significances between determination coefficients were obtained using t test; **, $p < 0.01$.

Figure S11

Scatter plot representing the relative enrichment of RNA in stress granules compared to the cytoplasm in cells with a normal (Si-Neg) or low (Si-FUS) FUS expression level. Significance was obtained using t test; ns: non significant.

Supplementary table

1: table of plasmids used for the MT bench bait-prey method

2: table of plasmids used for the MT bench compartmentalization method.

3: table of recombinant proteins produced for *in vitro* experiments and table of plasmids for production of recombinant proteins.

4: table of primary antibodies used for immunofluorescence experiences.

Supplementary Video

SV1: Three dimensional reconstruction of compiled confocal images of a large assembly of TDP-43 obtained after incubation for 2h.

SV2: Three dimensional reconstruction of compiled confocal images of assembly corresponding to Figure 4C obtained after incubation of FUS (in red) and TDP-43 (in green) at a FUS/TDP-43 molar ratio of 3/1 in presence of 2Luc mRNA (protein/nucleotide = 1/10) .

SV3: Same as SV2 with a TDP-43/FUS molar ratio of 9/1 (Figure 4D)

Calculation of the correlation coefficient from the bait/prey method (MT bench experiment)

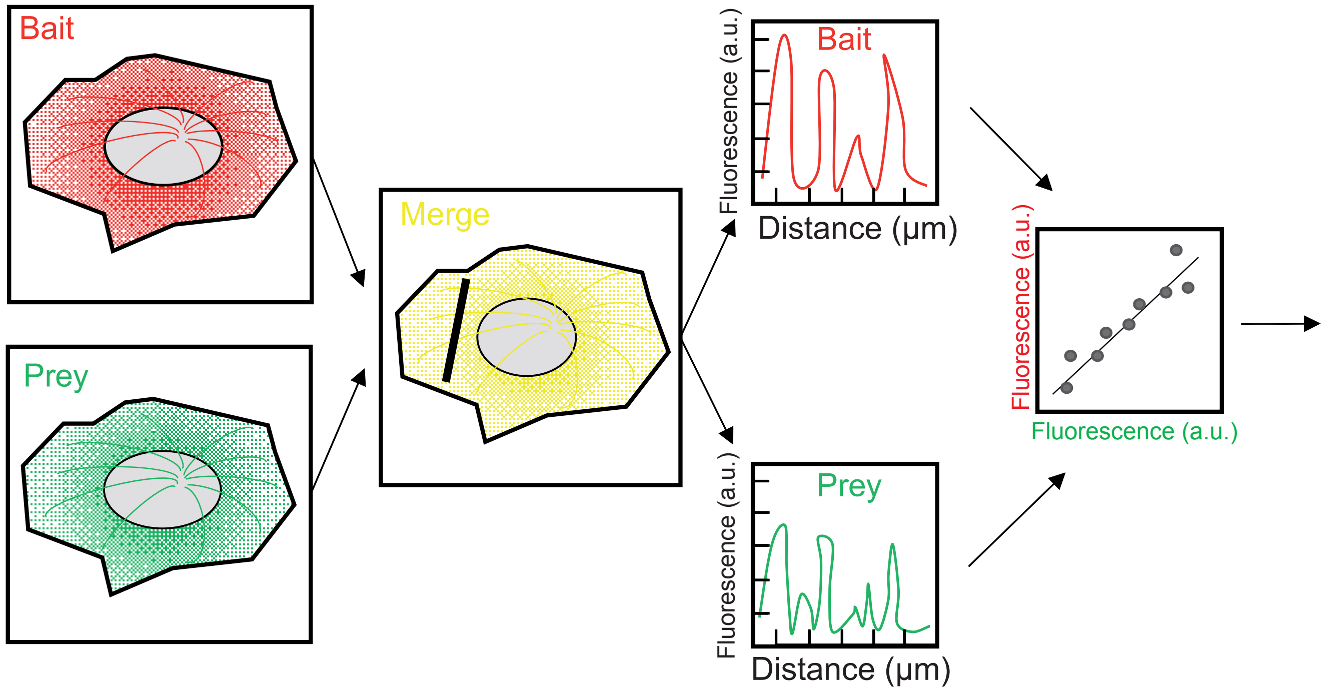

1. Aquisition of images of cells in red and green channels

2. A line crossing microtubules is drawn on merged image

3. Fluorescence intensity along the line is measured and transformed into numerical values

4. Pearson correlation coefficient is calculated for the two data sets

Supplementary figure S1

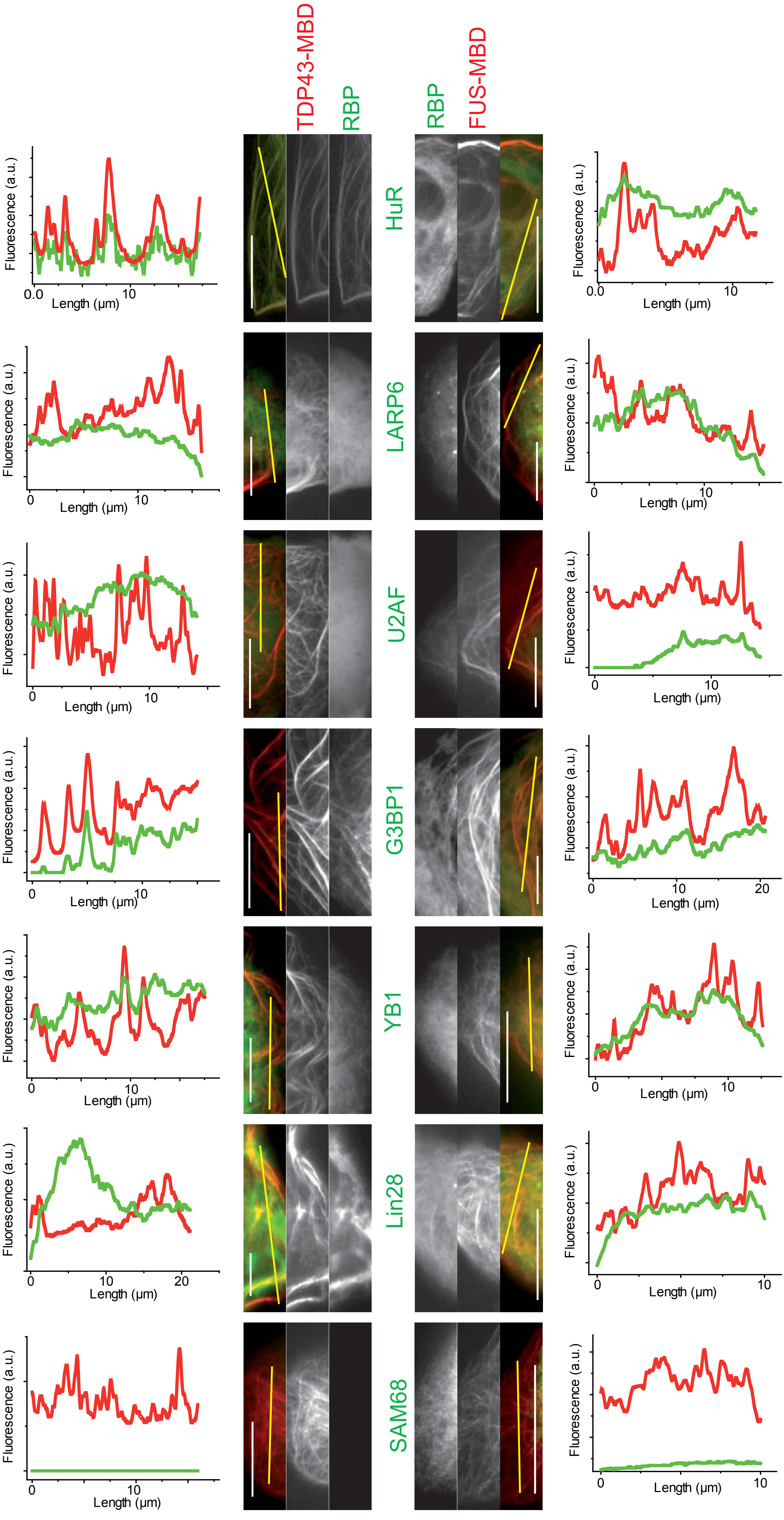

Supplementary figure S2

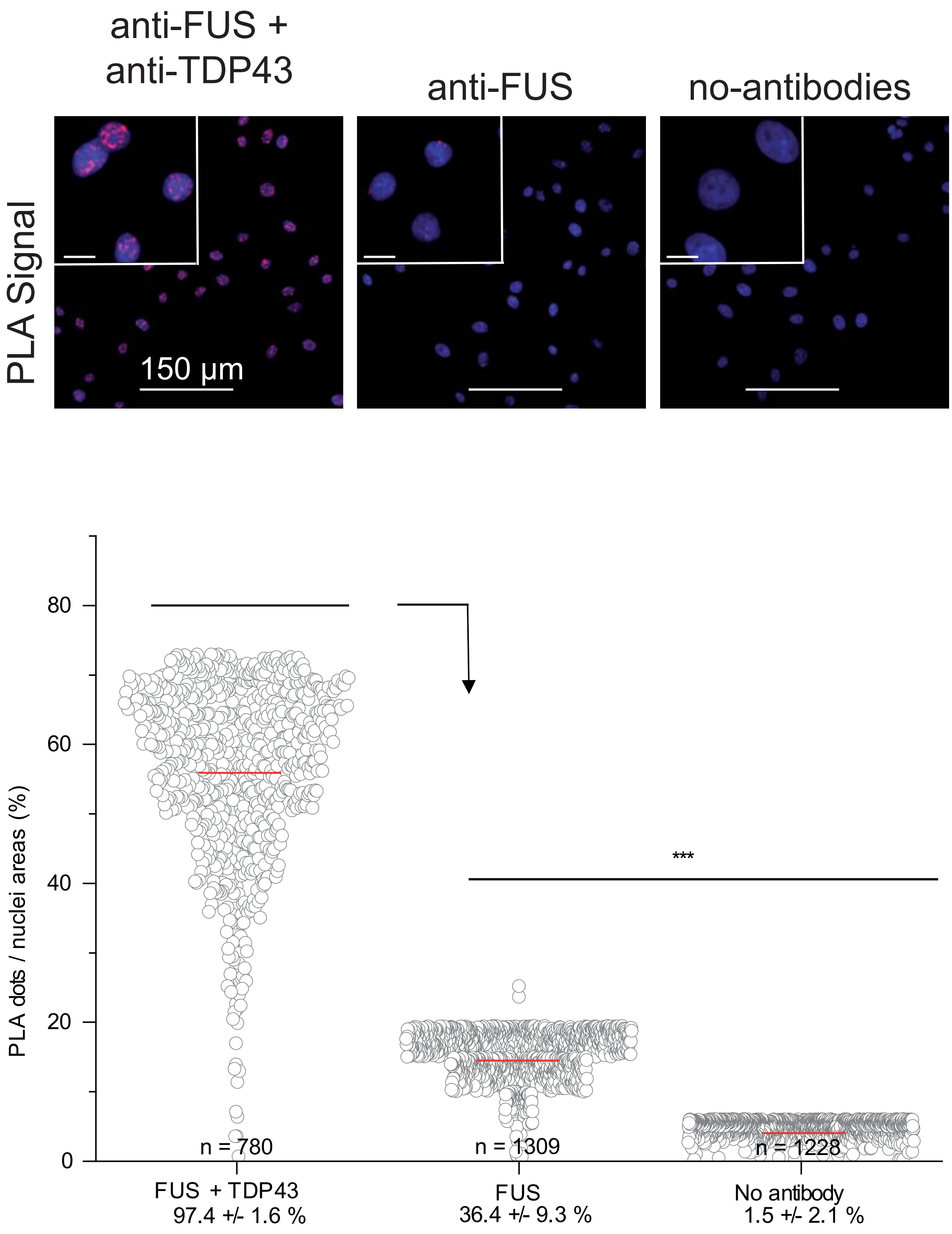

Supplementary Figure S3

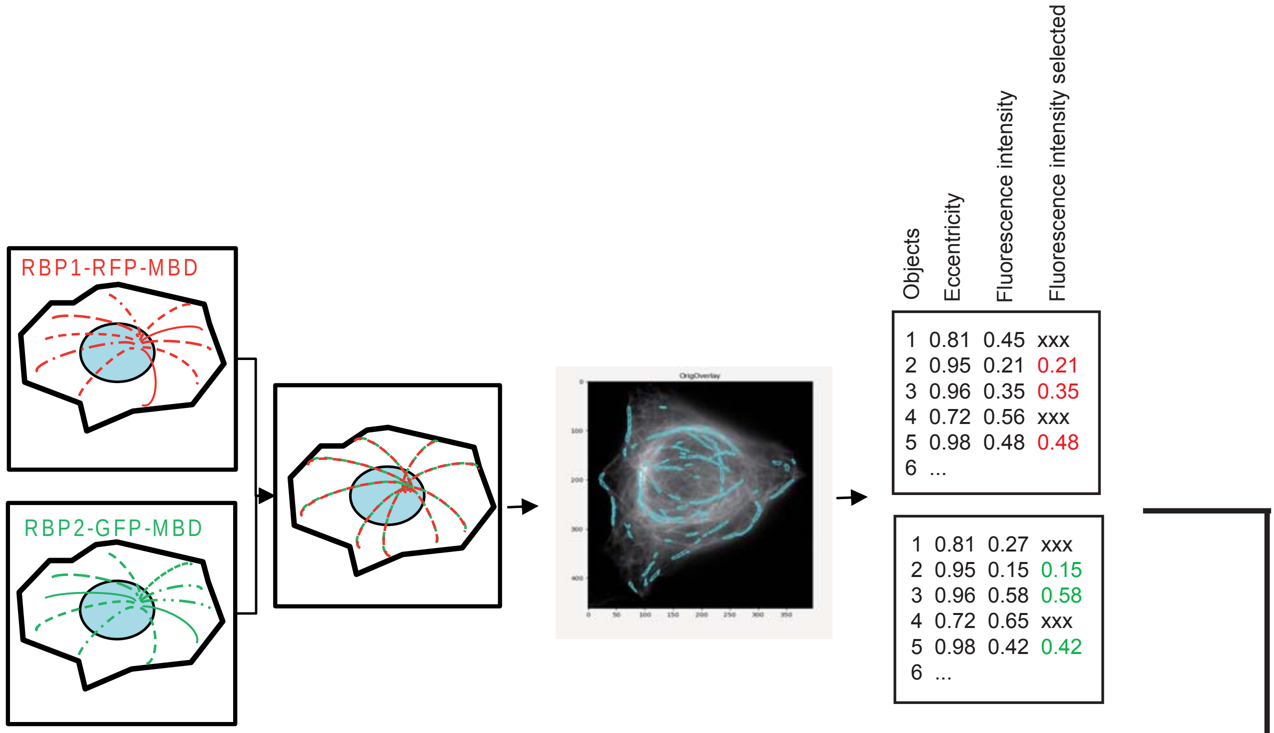

1- Images of cells transfected with RBP plasmids

2- On merged images, MT appeared as yellow (the RBPs mix) or with a green/red hatched pattern (no mix)

3- Program automaticaly detects MT and measures fluorescence intensities along them for each channel

4- Data are extracted, non tubular objects are discarded (eccentricity < 0.9) and background is substracted

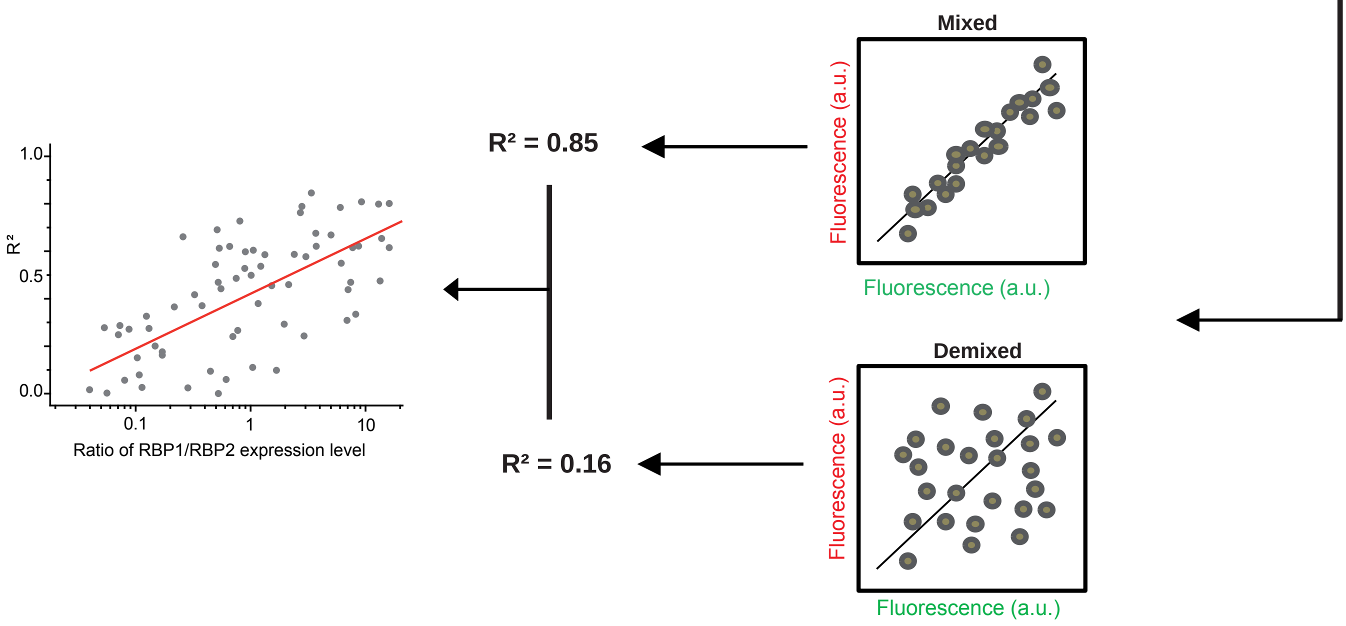

6-The R^2 are plotted for each cell according to the ratio of RBP1/RBP2 expression level

5- Determination of the determination coefficient R^2 from the plot of fluorescent intensities.

Supplementary Figure S4

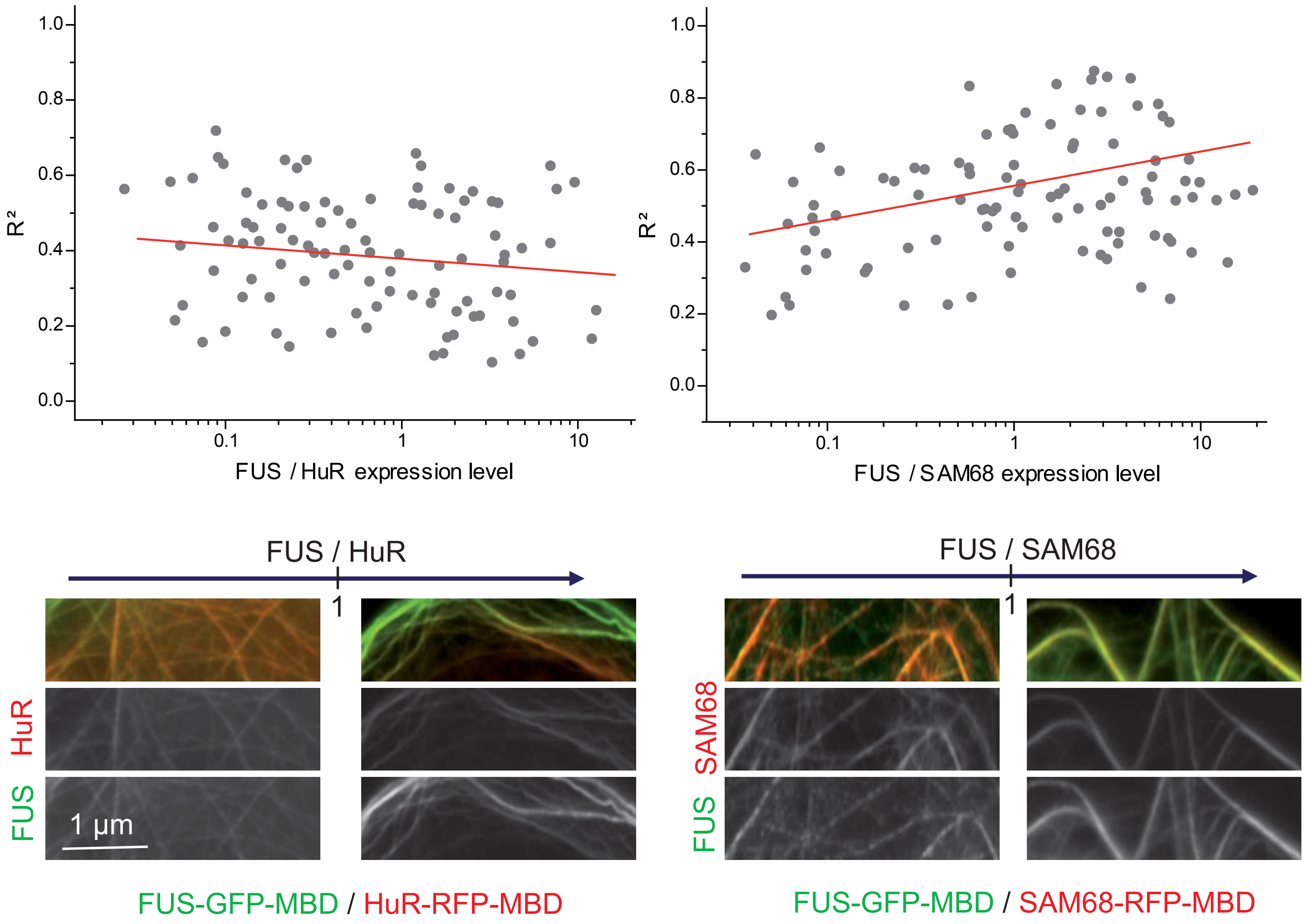

Supplementary figure S5

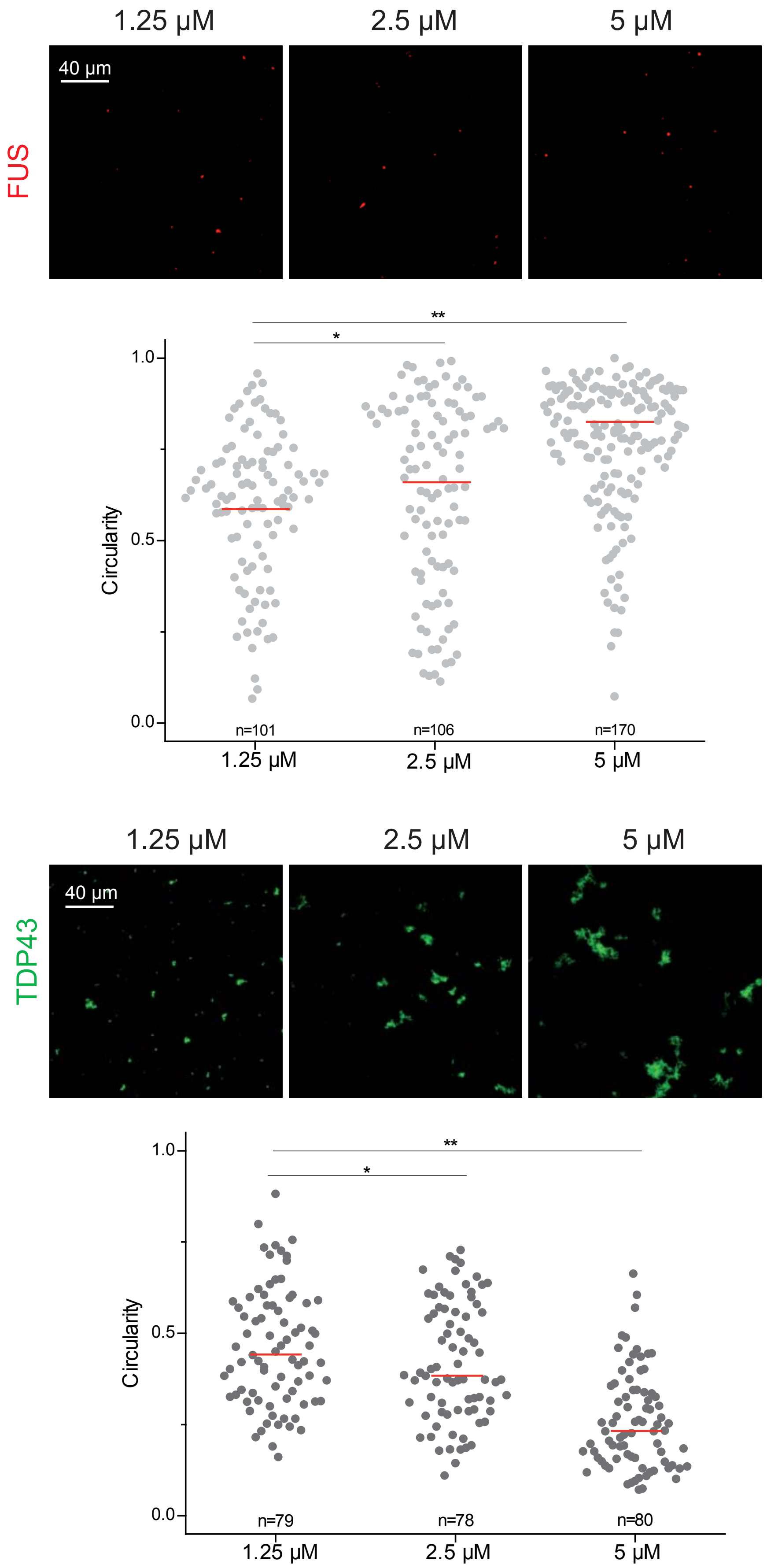

Supplementary Figure S6

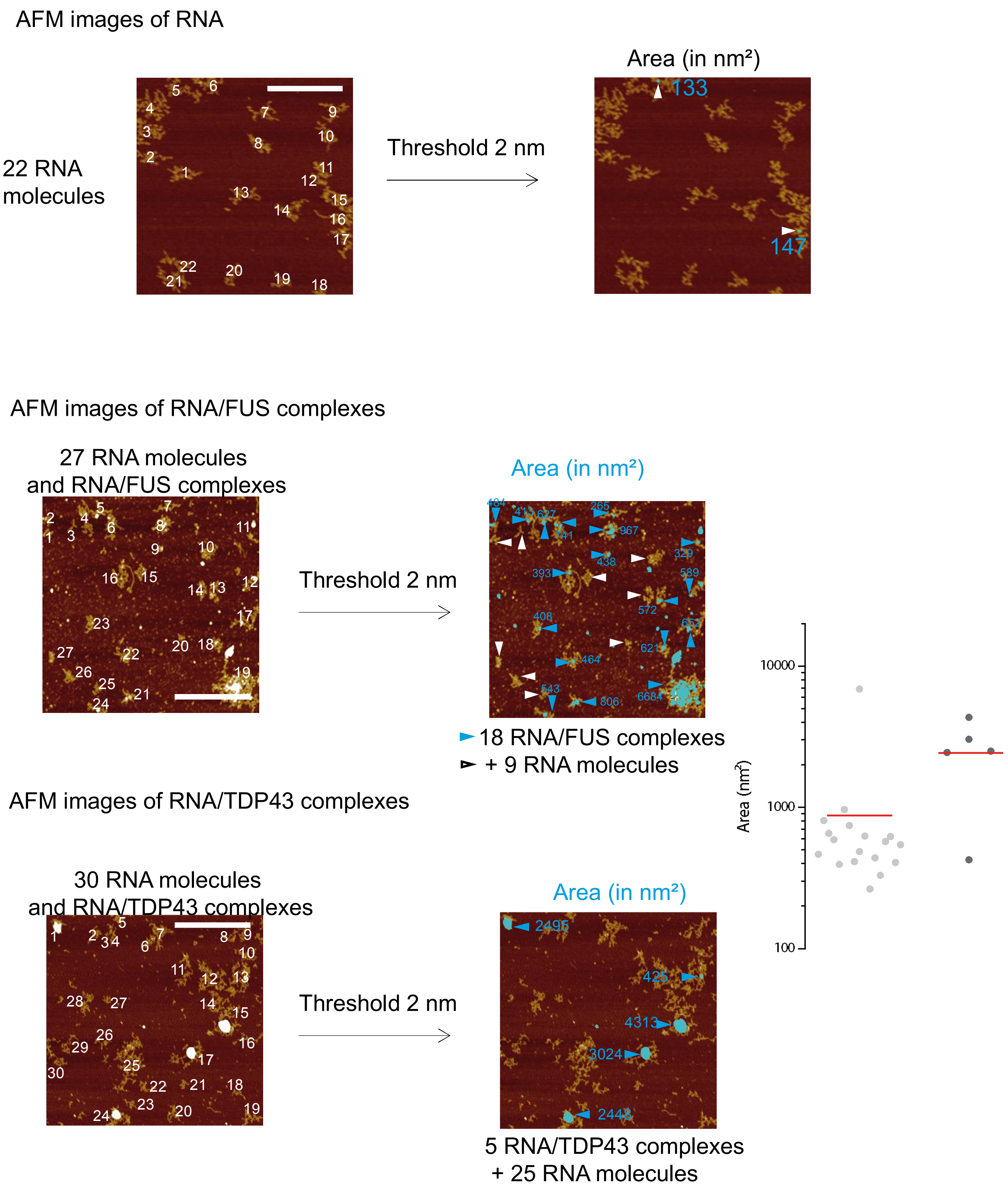

Supplementary figure S7

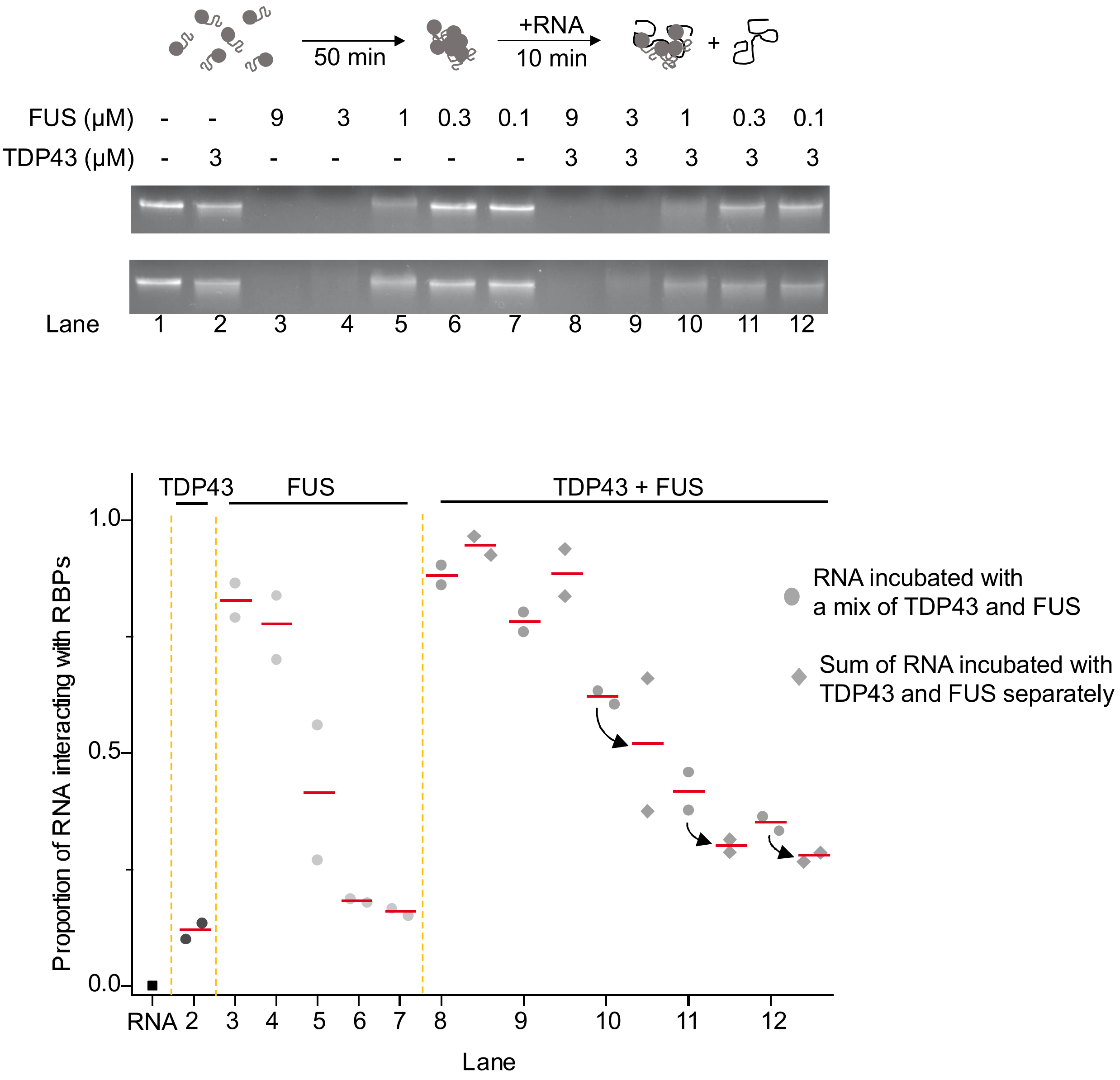

Supplementary Figure S8

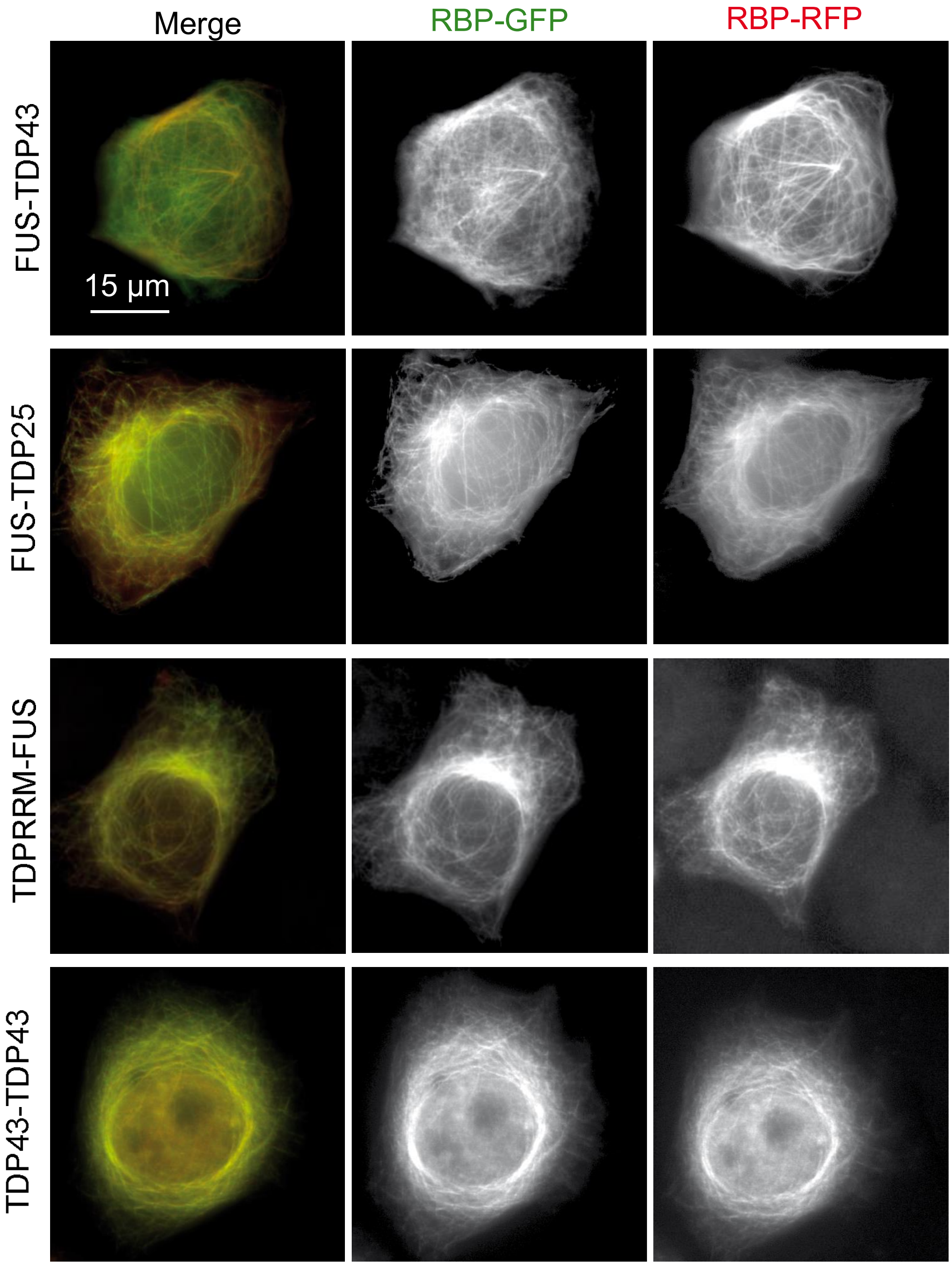

Supplementary figure S9

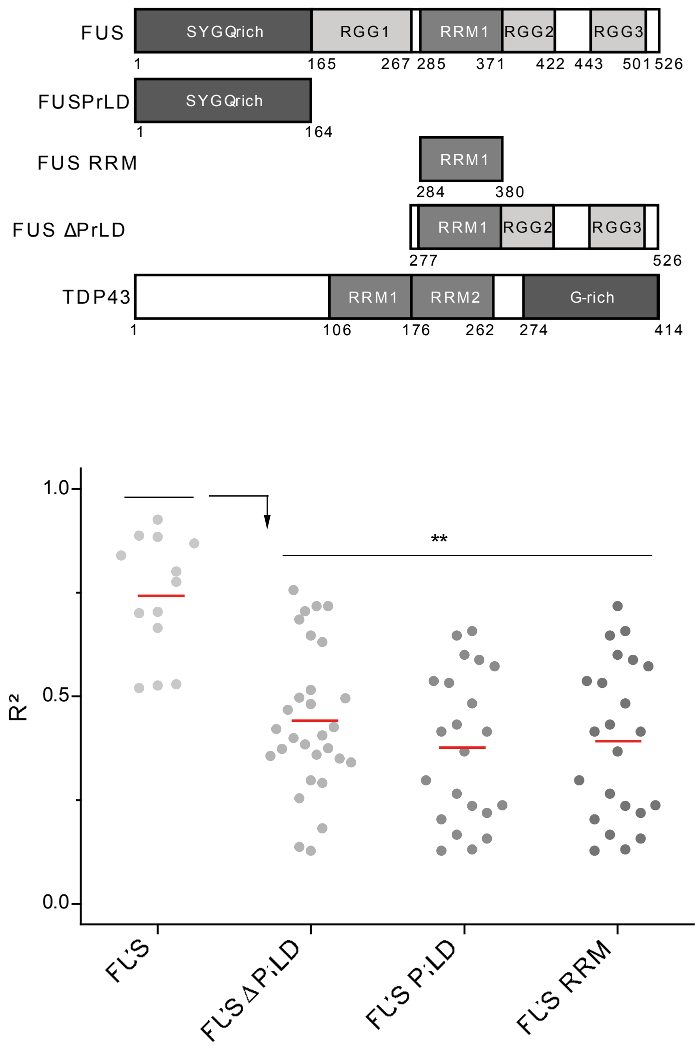

Supplementary figure S10

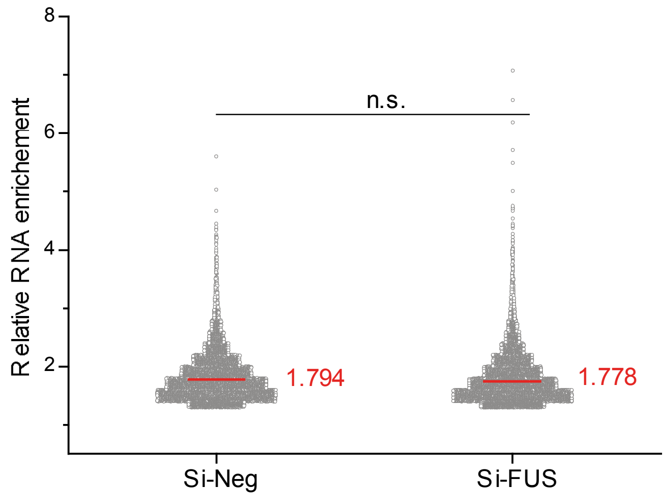

Supplementary Figure S11

Table of plasmids used for the Microtubule Bench bait-prey method

**Plasmid name**	**Sequence**	**tag**	**Primers or reference**
FUS-RFP-MBD	FL (1-526)	RFP-MBD	[1]
TDP43-RFP-MBD	FL (1-414)	RFP-MBD	[1]
FUS-GFP	FL (1-526)	GFP	[1]
TDP43-GFP	FL (1-414)	GFP	[1]
YB1-GFP	FL (1-324)	GFP	[2]
Lin28-GFP	FL (1-209)	GFP	[2]
HuR-GFP	FL (1-326)	GFP	[1]
G3BP-GFP	FL (1-466)	GFP	[2]
Larp6-GFP	FL (1-491)	GFP	[2]
U2AF-GFP	FL (1-475)	GFP	[1]
Sam68-GFP	FL (1-443)	GFP	[3]

Supplementary table 1

Table of plasmids used for the Microtubule Bench compartmentalization method

**Name**	**Sequence**	**Primers or reference**
FUS-GFP-MBD	FL (1-526)	[1]
FUS-RFP-MBD	FL (1-526)	[1]
TDP43-GFP-MBD	FL (1-414)	[1]
TDP43-RFP-MBD	FL (1-414)	[1]
Sam68-RFP-MBD	FL (1-443)	[3]
HuR-RFP-MBD	FL (1-326)	[1]
TDP RRM-GFP-MBD	98-263	GCGTTAATTAAGCCACCATGGCAGTCCAGAAAACATCCGAT /GCGGGCGCGCCTGGTGCTTAGGTTCGGCATTGGA
TDP Nter-GFP-MBD	1-102	GCGTTAATTAAGCCACCATGTCTGAATATATTCGGGTAACCG/GCGGGCGCGCCTGTGTTTCTGGACTGCTCTTTTCAC
FUS RRM-GPF-MBD	284-380	GCGTTAATTAAATGAATTCAGACAACAACACCATC /GCGGGCGCGCCTTGCGAGTAGCAAATGAGACCTTG
FUS PrLD-GFP-MBD	1-164	GCGTTAATTAAGCCATGGCCTCAAACGATTATACCC/ GCGGGCGCGCCatACTGCTGCTGTTGTACTGGTT
FUS ΔLCD	277-525	[1]
TDP ΔPrLD-RFP-MBD	1-277	[1]
TDP25-RFP-MBD	208-414	GCGTTAATTAAATGCGGGAGTTCTTCTCTCAGTACGGGG/ GCGGGCGCGCCATCATTCCCCAGCCAGAAGACTTAGAA
TDP35-RFP-MBD	102-414	GCGTTAATTAAATGACGGAATATGAAACACAAGTGAAAG /GCGGGCGCGCCATCATTCCCCAGCCAGAAGACTTAGAA
TDP PrLD	270-414	[1]
mCherry-MBD	FL	[2]

Supplementary table 2

Table of recombinant proteins (full length and truncated) produced for *in vitro* experiments.

**Protein name**	**Tag**	**Sequence**	**Molecular Weight (kDa)**
**TDP43**	**6His**	**1-414**	**44**
TDP RRM	6His-HA	98-213	20
TDP25	6His-HA	208-414	25
**FUS**	**6His**	**1-526**	**55**

Table of plasmid for production of recombinant proteins (truncated forms).

**Name**	**Restriction sites**	**Primers forward**	**Primers reverse**
TDP RRM	Nhe1/BamH1	GCGGCTAGCGGCAGTCCAGAAAAC ATCCGA	GCGGGATCCGTGCTTAGGTTCG GCATTGG
	HindIII/Xho1	GCGAAGCTTATGGGCTACCCCTACG ACGTG	GCGCTCGAGGTGCTTAGGTTCG GCATTGG
TDP25	Nhe1/BamH1	GCGGCTAGCGCGGGAGTTCTTCTCT CAGTA	CGCGGATCCCATTCCCCAGCCA GAAGACTTAGA
	HindIII/Xho1	GCG AAGCTTATGGGCTACCCCTACGACG TG	CTCGAGCATTCCCCAGCCAGAA GACTTAGA

Supplementary Table 3

Table of primary antibodies used for immunofluorescence experiences

Antibodies	Species	Epitope	Reference
α-TDP43	Mouse	AA 1-260 (Nter)	mAB ABnova H00023435-M01
α-TDP43	Rabbit	(Cter)	pAB Protein Teck 12892
α-FUS	Mouse	YGGQQQSYGQ (PrLD)	Novus CLO 190 mAB-03309
α-FUS	Rabbit	AA 1-50 (PrLD)	Novus Bio pAB NB 100-565
α-HA	Rabbit	Tag HA	H6908 Sigma
α-Myc	Mouse	Tag Myc	sAB1305535 Sigma (9E10)
α-Dig	Chicken	Tag Dig	pAb AB51946 Abcam

Supplementary table 4

[1] Maucuer A, Desforges B, Joshi V, Boca M, Kretov D, Hamon L, et al. Microtubules as platforms for probing liquid-liquid phase separation in cells: application to RNA-binding proteins. Journal of cell science 2018.
[2] Boca M, Kretov DA, Desforges B, Mephon-Gaspard A, Curmi PA, Pastre D. Probing protein interactions in living mammalian cells on a microtubule bench. Scientific reports 2015;5:17304.
[3] Pankivskyi S, Pastre D, Steiner E, Joshi V, Rynditch A, Hamon L. ITSN1 regulates SAM68 solubility through SH3 domain interactions with SAM68 proline-rich motifs. Cellular and molecular life sciences : CMLS 2020.
